# Supplementary material for: Korean Medicine Clinical Practice Guidelines for Lumbar Herniated Intervertebral Disc in Adults: Based on Grading of Recommendations Assessment, Development and Evaluation (GRADE)
Source: Healthcare (Basel). 2022 Jan 27;10(2):246. doi: 10.3390/healthcare10020246 (PMC8871943; doi:10.3390/healthcare10020246)
Supplement: Supplementary file 1 [file healthcare-10-00246-s001.zip › Supplementary S1.pdf]

**Search strategy for the Patients/Populations and Intervention components**

**P: Lumbar HIVD patients(adults)**

**I: Acupuncture, Moxibustion, Herbal medicine, Pharmacopuncture, Chuna manual therapy, Thread-embedding acupuncture, Cupping / (Monotherapy, Combination therapy, Specific technique of Korean medicine treatment)**

**C: Active control Group, Conventional treatment, other technique of treatment same as intervention group.**

**O: Total clinical effective rate(Number of subjects who showed improvement in symptoms), pain(VAS), Function(JOA, m-JOA, ODI)**

**1) Acupuncture**

**(1) English**

**① MEDLINE via Pubmed**

#1 Intervertebral Disc Displacement[Mesh]

#2 Sciatica[Mesh]

#3 Polyradiculopathy[Mesh]

#4 (disc OR discs OR disk OR disks OR nucleus pulposus OR sacroilia* OR Sacroiliac-joint)

#5 (displacement OR degeneration OR hernia* OR protru* OR perfora* OR ruptur* OR degenerat* OR degradat* OR displac* OR prolaps* OR avuls* OR extru*)

#6 #4 AND #5

#7 (nerve root OR nerve roots OR nerve)

#8 (compress* OR entrap* OR inflammat* OR disorder*)

#9 #7 AND #8

#10 (polyradiculopathy OR radiculopath* OR radiculiti* OR sciatic)

#11 {or #1, #2, #3, #6, #9, #10}

#12 (Acupuncture)

#13 #11 AND #12

**② CENTRAL via Cochrane**

#1 Intervertebral disk displacement[ti,ab,kw]

#2 sciatica[ti,ab,kw]

#3 polyradiculopathy[ti,ab,kw]

#4 (disc OR discs OR disk OR disks OR nucleus pulposus OR sacroilia* OR Sacroiliac-joint)[ti,ab,kw]

#5 (displacement OR degeneration OR hernia* OR protru* OR perfora* OR ruptur* OR degenerat* OR degradat* OR displac* OR prolaps* OR avuls* OR extru* )[ti,ab,kw]

#6 #4 AND #5

#7 (nerve root OR nerve roots OR nerve)[ti,ab,kw]

#8 (compress* OR entrap* OR inflammat* OR disorder*)[ti,ab,kw]

#9 #7 AND #8

#10 (polyradiculopathy OR radiculopath* OR radiculiti* OR sciatic)[ti,ab,kw]

#11 {or #1-#3, #6, #9, #10}

#12 (Acupuncture)[ti,ab,kw]

#13 #11 AND #12

③ **EMBASE**

#1 'Intervertebral disk displacement'/exp

#2 'sciatica'/exp

#3 'polyradiculopathy'/exp

#4 (disc OR discs OR disk OR disks OR nucleus pulposus OR sacroilia* OR Sacroiliac-joint):ab,ti

#5 (displacement OR degeneration OR hernia* OR protru* OR perfora* OR ruptur* OR degenerat* OR degradat* OR displac* OR prolaps* OR avuls* OR extru* ):ab,ti

#6 #4 AND #5

#7 (nerve root OR nerve roots OR nerve):ab,ti

#8 (compress* OR entrap* OR inflammat* OR disorder*):ab,ti

#9 #7 AND #8

#10 (polyradiculopathy OR radiculopath* OR radiculiti* OR sciatic):ab,ti

#11 {or #1-#3, #6, #9, #10}

#12 (Acupuncture):ab,ti

#13 #11 AND #12

**(2) Chinese**

① **CNKI**

“HIVD” OR “Herniated Intervertebral disc” OR “HNP” OR “Herniated nucleus pulposus” OR “spinal disc herniation” OR “intervertebral disc herniation” OR “herniated disc” OR “Intervertebral disc displacement”

AND

"Acupuncture"

**(3) Japansese**

**① CiNII and J-Stage**

((((((((椎間板 OR 神経根症 OR ディスク) OR (Intervertebral Disc Displacement OR Sciatica) OR Polyradiculopathy) OR ((((disc OR discs OR disk OR disks OR nucleus pulposus OR sacroilia* OR Sacroiliac-joint))) AND ((displacement OR degeneration OR hernia* OR protru* OR perfora* OR ruptur* OR degenerat* OR degradat* OR displac* OR prolaps* OR avuls* OR extru*)))) OR ((((nerve root OR nerve roots OR nerve))) AND ((compress* OR entrap* OR inflammat* OR disorder*)))) OR (((polyradiculopathy OR radiculopath* OR radiculiti* OR sciatic))))

AND (Acupuncture OR しん治療 OR しん療法 OR しん術 OR はり刺激 OR はり治療 OR はり療法 OR 刺しん法 OR 刺しん術 OR 針 OR 鍼)

**(4) Korean**

**① KoreaMed**

((disc[ALL] OR nucleus pulposus[ALL]) AND (displacement[ALL] OR degeneration[ALL] OR hernia[ALL] ))

AND

(Acupuncture[ALL])

**② KMbase**

((([ALL=disc] OR [ALL=nucleus pulposus]) AND ([AHLL=displacement] OR [ALL=degeneration] OR [ALL=hernia*])) OR [ALL=추간판 탈출증] OR [ALL=디스크 탈출증]) AND ([ALL=침] OR [ALL=Acupuncture])

**③ KISS**

(((disc OR nucleus pulposus) AND (displacement OR degeneration OR hernia*)) OR (추간판 탈출증 OR 디스크 탈출증) AND (침 OR Acupuncture)

④ **NDSL**

(((disc OR nucleus pulposus) AND (displacement OR degeneration OR hernia*)) OR (추간판 탈출증 OR 디스크 탈출증) AND (침 OR Acupuncture)

⑤ **KISTI**

(BI: disc hernia*) OR (BI: nucleus pulposus hernia*) OR (BI: disc displacement) OR (BI: nucleus pulposus displacement) OR (BI: disc degeneration) OR (BI: nucleus pulposus degeneration) OR (BI: 추간판 탈출증) OR (BI: 디스크 탈출증)

AND

(BI: 침) OR (BI: Acupuncture)

⑥ **OASIS**

(((disc OR nucleus pulposus) AND (displacement OR degeneration OR hernia*)) OR (추간판 탈출증 OR 디스크 탈출증) AND (침 OR Acupuncture)

**2) Moxibustion**

**(1) English**

① **MEDLINE via Pubmed**

#1 Intervertebral Disc Displacement[Mesh]

#2 Sciatica[Mesh]

#3 Polyradiculopathy[Mesh]

#4 (disc OR discs OR disk OR disks OR nucleus pulposus OR sacroilia* OR Sacroiliac-joint)

#5 (displacement OR degeneration OR hernia* OR protru* OR perfora* OR ruptur* OR degenerat* OR degradat* OR displac* OR prolaps* OR avuls* OR extru*)

#6 #4 AND #5

#7 (nerve root OR nerve roots OR nerve)

#8 (compress* OR entrap* OR inflammat* OR disorder*)

#9 #7 AND #8

#10 (polyradiculopathy OR radiculopath* OR radiculiti* OR sciatic)

#11 {or #1, #2, #3, #6, #9, #10}

#12 (Moxibustion)

#13 #11 AND #12

② **CENTRAL via Cochrane**

#1 Intervertebral disk displacement[ti,ab,kw]

#2 sciatica[ti,ab,kw]

#3 polyradiculopathy[ti,ab,kw]

#4 (disc OR discs OR disk OR disks OR nucleus pulposus OR sacroilia* OR Sacroiliac-joint)[ti,ab,kw]

#5 (displacement OR degeneration OR hernia* OR protru* OR perfora* OR ruptur* OR degenerat* OR degradat* OR displac* OR prolaps* OR avuls* OR extru* )[ti,ab,kw]

#6 #4 AND #5

#7 (nerve root OR nerve roots OR nerve)[ti,ab,kw]

#8 (compress* OR entrap* OR inflammat* OR disorder*)[ti,ab,kw]

#9 #7 AND #8

#10 (polyradiculopathy OR radiculopath* OR radiculiti* OR sciatic)[ti,ab,kw]

#11 {or #1-#3, #6, #9, #10}

#12 (Moxibustion)[ti,ab,kw]

#13 #11 AND #12

③ **EMBASE**

#1 ‘Intervertebral disk displacement’/exp

#2 ‘sciatica’/exp

#3 ‘polyradiculopathy’/exp

#4 (disc OR discs OR disk OR disks OR nucleus pulposus OR sacroilia* OR Sacroiliac-joint):ab,ti

#5 (displacement OR degeneration OR hernia* OR protru* OR perfora* OR ruptur* OR degenerat* OR degradat* OR displac* OR prolaps* OR avuls* OR extru* ):ab,ti

#6 #4 AND #5

#7 (nerve root OR nerve roots OR nerve):ab,ti

#8 (compress* OR entrap* OR inflammat* OR disorder*):ab,ti

#9 #7 AND #8

#10 (polyradiculopathy OR radiculopath* OR radiculiti* OR sciatic):ab,ti

#11 {or #1-#3, #6, #9, #10}

#12 (Moxibustion):ab,ti

#13 #11 AND #12

**(2) chinese**

① **CNKI**

“HIVD” OR “Herniated Intervertebral disc” OR “HNP” OR “Herniated nucleus pulposus” OR “spinal disc herniation” OR “intervertebral disc herniation” OR “herniated disc” OR “Intervertebral disc displacement”

AND

"Moxibustion“

**(3) Japanese**

① **CiNII and J-Stage**

((((((((椎間板 OR 神経根症 OR ディスク) OR (Intervertebral Disc Displacement OR Sciatica) OR Polyradiculopathy) OR ((((disc OR discs OR disk OR disks OR nucleus pulposus OR sacroilia* OR Sacroiliac-joint))) AND ((displacement OR degeneration OR hernia* OR protru* OR perfora* OR ruptur* OR degenerat* OR degradat* OR displac* OR prolaps* OR avuls* OR extru*)))) OR ((((nerve root OR nerve roots OR nerve))) AND ((compress* OR entrap* OR inflammat* OR disorder*)))) OR (((polyradiculopathy OR radiculopath* OR radiculiti* OR sciatic))))

AND (Moxibustion OR 灸)

**(4) Korean**

① **KoreaMed**

((disc[ALL] OR nucleus pulposus[ALL]) AND (displacement[ALL] OR degeneration[ALL] OR hernia[ALL] ))

AND

(뜸[ALL] OR Moxibustion[ALL])

② **KMbase**

(((([ALL=disc] OR [ALL=nucleus pulposus]) AND ([AHLL=displacement] OR [ALL=degeneration] OR [ALL=hernia*])) OR [ALL=추간판 탈출증] OR [ALL=디스크 탈출증]) AND ([ALL=뜸] OR [ALL=Moxibustion])

③ **KISS**

(((disc OR nucleus pulposus) AND (displacement OR degeneration OR hernia*)) OR (추간판 탈출증 OR 디스크 탈출증) AND (뜸 OR Moxibustion)

④ **NDSL**

(((disc OR nucleus pulposus) AND (displacement OR degeneration OR hernia*)) OR (추간판 탈출증 OR 디스크 탈출증) AND (뜸 OR Moxibustion)

⑤ **KISTI**

(BI: disc hernia*) OR (BI: nucleus pulposus hernia*) OR (BI: disc displacement) OR (BI: nucleus pulposus displacement) OR (BI: disc degeneration) OR (BI: nucleus pulposus degeneration) OR (BI: 추간판 탈출증) OR (BI: 디스크 탈출증)

AND

(BI: 뜸) OR (BI: Moxibustion)

⑥ **OASIS**

(((disc OR nucleus pulposus) AND (displacement OR degeneration OR hernia*)) OR (추간판 탈출증 OR 디스크 탈출증) AND (뜸 OR Moxibustion)

**3) Herbal Medicine**

**(1) English**

① **MEDLINE via Pubmed**

#1 Intervertebral Disc Displacement[Mesh]

#2 Sciatica[Mesh]

#3 Polyradiculopathy[Mesh]

#4 (disc OR discs OR disk OR disks OR nucleus pulposus OR sacroilia* OR Sacroiliac-joint)

#5 (displacement OR degeneration OR hernia* OR protru* OR perfora* OR ruptur* OR degenerat* OR degradat* OR displac* OR prolaps* OR avuls* OR extru*)

#6 #4 AND #5

#7 (nerve root OR nerve roots OR nerve)

#8 (compress* OR entrap* OR inflammat* OR disorder*)

#9 #7 AND #8

#10 (polyradiculopathy OR radiculopath* OR radiculiti* OR sciatic)

#11 {or #1, #2, #3, #6, #9, #10}

#12 (Herb medicine OR Decoction)

#13 #11 AND #12

② **CENTRAL via Cochrane**

#1 Intervertebral disk displacement[ti,ab,kw]

#2 sciatica[ti,ab,kw]

#3 polyradiculopathy[ti,ab,kw]

#4 (disc OR discs OR disk OR disks OR nucleus pulposus OR sacroilia* OR Sacroiliac-joint)[ti,ab,kw]

#5 (displacement OR degeneration OR hernia* OR protru* OR perfora* OR ruptur* OR degenerat* OR degradat* OR displac* OR prolaps* OR avuls* OR extru* )[ti,ab,kw]

#6 #4 AND #5

#7 (nerve root OR nerve roots OR nerve)[ti,ab,kw]

#8 (compress* OR entrap* OR inflammat* OR disorder*)[ti,ab,kw]

#9 #7 AND #8

#10 (polyradiculopathy OR radiculopath* OR radiculiti* OR sciatic)[ti,ab,kw]

#11 {or #1-#3, #6, #9, #10}

#12 (Herb medicine OR Decoction)[ti,ab,kw]

#13 #11 AND #12

③ **EMBASE**

#1 ‘Intervertebral disk displacement’/exp

#2 ‘sciatica’/exp

#3 ‘polyradiculopathy’/exp

#4 (disc OR discs OR disk OR disks OR nucleus pulposus OR sacroilia* OR Sacroiliac-joint):ab,ti

#5 (displacement OR degeneration OR hernia* OR protru* OR perfora* OR ruptur* OR degenerat* OR degradat* OR displac* OR prolaps* OR avuls* OR extru* ):ab,ti

#6 #4 AND #5

#7 (nerve root OR nerve roots OR nerve):ab,ti

#8 (compress* OR entrap* OR inflammat* OR disorder*):ab,ti

#9 #7 AND #8

#10 (polyradiculopathy OR radiculopath* OR radiculiti* OR sciatic):ab,ti

#11 {or #1-#3, #6, #9, #10}

#12 (Herb medicine OR Decoction):ab,ti

#13 #11 AND #12

**(2) Chinese**

① **CNKI**

“HIVD” OR “Herniated Intervertebral disc” OR “HNP” OR “Herniated nucleus pulposus” OR “spinal disc herniation” OR “intervertebral disc herniation” OR “herniated disc” OR “Intervertebral disc displacement”

AND

"Herb medicine" OR “Decoction”

**(3) Japanese**

① **CiNII and J-Stage**

((((((((椎間板 OR 腰部神経根症) OR (Intervertebral Disc Displacement OR Sciatica) OR Polyradiculopathy) OR ((((disc OR discs OR disk OR disks OR nucleus pulposus OR sacroilia* OR Sacroiliac-joint))) AND ((displacement OR degeneration OR hernia* OR protru* OR perfora* OR ruptur* OR degenerat* OR degradat* OR displac* OR prolaps* OR avuls* OR extru*)))) OR ((((nerve root OR nerve roots OR nerve))) AND ((compress* OR entrap* OR inflammat* OR disorder*)))) OR (((polyradiculopathy OR radiculopath* OR radiculiti* OR sciatic))))

AND (Japanese medicine OR 日本薬 OR 漢方 OR 本草OR 漢方薬 OR 漢薬 OR KAMPO OR herb OR

herbal medicine OR decoction OR herb medicine)

**(4) Korean**

① **KoreaMed**

((disc[ALL] OR nucleus pulposus[ALL]) AND (displacement[ALL] OR degeneration[ALL] OR hernia[ALL] ))
AND
(한약[ALL] OR Herb medicine[ALL])

② **KMbase**

((([ALL=disc] OR [ALL=nucleus pulposus]) AND ([AHLL=displacement] OR [ALL=degeneration] OR [ALL=hernia*])) OR [ALL=추간판 탈출증] OR [ALL=디스크 탈출증]) AND ([ALL=한약] OR [ALL=Herb medicine])

③ **KISS**

(((disc OR nucleus pulposus) AND (displacement OR degeneration OR hernia*)) OR (추간판 탈출증 OR 디스크 탈출증) AND (한약 OR Herb medicine)

④ **NDSL**

(((disc OR nucleus pulposus) AND (displacement OR degeneration OR hernia*)) OR (추간판 탈출증 OR 디스크 탈출증) AND (한약 OR Herb medicine)

⑤ **KISTI**

(BI: disc hernia*) OR (BI: nucleus pulposus hernia*) OR (BI: disc displacement) OR (BI: nucleus pulposus displacement) OR (BI: disc degeneration) OR (BI: nucleus pulposus degeneration) OR (BI: 추간판 탈출증) OR (BI: 디스크 탈출증)
AND
(BI: 한약) OR (BI: Herb medicine)

⑥ **OASIS**

(((disc OR nucleus pulposus) AND (displacement OR degeneration OR hernia*)) OR (추간판 탈출증 OR 디스크 탈출증) AND (한약 OR Herb medicine)

**4) Pharmacopuncture**

**(1) English**

**① MEDLINE via Pubmed**

#1 Intervertebral Disc Displacement[Mesh]

#2 Sciatica[Mesh]

#3 Polyradiculopathy[Mesh]

#4 (disc OR discs OR disk OR disks OR nucleus pulposus OR sacroilia* OR Sacroiliac-joint)

#5 (displacement OR degeneration OR hernia* OR protru* OR perfora* OR ruptur* OR degenerat* OR degradat* OR displac* OR prolaps* OR avuls* OR extru*)

#6 #4 AND #5

#7 (nerve root OR nerve roots OR nerve)

#8 (compress* OR entrap* OR inflammat* OR disorder*)

#9 #7 AND #8

#10 (polyradiculopathy OR radiculopath* OR radiculiti* OR sciatic)

#11 {or #1, #2, #3, #6, #9, #10}

#12 (Pharmacoacupuncture OR Bee venom)

#13 #11 AND #12

**② CENTRAL via Cochrane**

#1 Intervertebral disk displacement[ti,ab,kw]

#2 sciatica[ti,ab,kw]

#3 polyradiculopathy[ti,ab,kw]

#4 (disc OR discs OR disk OR disks OR nucleus pulposus OR sacroilia* OR Sacroiliac-joint)[ti,ab,kw]

#5 (displacement OR degeneration OR hernia* OR protru* OR perfora* OR ruptur* OR degenerat* OR degradat* OR displac* OR prolaps* OR avuls* OR extru* )[ti,ab,kw]

#6 #4 AND #5

#7 (nerve root OR nerve roots OR nerve)[ti,ab,kw]

#8 (compress* OR entrap* OR inflammat* OR disorder*)[ti,ab,kw]

#9 #7 AND #8

#10 (polyradiculopathy OR radiculopath* OR radiculiti* OR sciatic)[ti,ab,kw]

#11 {or #1-#3, #6, #9, #10}

#12 (Pharmacoacupuncture OR Bee venom)[ti,ab,kw]

#13 #11 AND #12

**③ EMBASE**

#1 'Intervertebral disk displacement'/exp

#2 'sciatica'/exp

#3 'polyradiculopathy'/exp

#4 (disc OR discs OR disk OR disks OR nucleus pulposus OR sacroilia* OR Sacroiliac-joint):ab,ti

#5 (displacement OR degeneration OR hernia* OR protru* OR perfora* OR ruptur* OR degenerat* OR degradat* OR displac* OR prolaps* OR avuls* OR extru* ):ab,ti

#6 #4 AND #5

#7 (nerve root OR nerve roots OR nerve):ab,ti

#8 (compress* OR entrap* OR inflammat* OR disorder*):ab,ti

#9 #7 AND #8

#10 (polyradiculopathy OR radiculopath* OR radiculiti* OR sciatic):ab,ti

#11 {or #1-#3, #6, #9, #10}

#12 (Pharmacoacupuncture OR Bee venom):ab,ti

#13 #11 AND #12

**(2) Chinese**

**① CNKI**

"HIVD" OR "Herniated Intervertebral disc" OR "HNP" OR "Herniated nucleus pulposus" OR "spinal disc herniation" OR "intervertebral disc herniation" OR "herniated disc" OR "Intervertebral disc displacement"

AND

"Pharmacoacupuncture" OR "Bee venom"

**(3) Japanese**

① **CiNII and J-Stage**

((((((((椎間板 OR 神経根症 OR ディスク) OR (Intervertebral Disc Displacement OR Sciatica) OR Polyradiculopathy) OR ((((disc OR discs OR disk OR disks OR nucleus pulposus OR sacroilia* OR Sacroiliac-joint))) AND ((displacement OR degeneration OR hernia* OR protru* OR perfora* OR ruptur* OR degenerat* OR degradat* OR displac* OR prolaps* OR avuls* OR extru*)))) OR ((((nerve root OR nerve roots OR nerve))) AND ((compress* OR entrap* OR inflammat* OR disorder*)))) OR (((polyradiculopathy OR radiculopath* OR radiculiti* OR sciatic))))

AND (Pharmacoacupuncture OR Bee venom OR 藥鍼)

**(4) Korean**

① **KoreaMed**

((disc[ALL] OR nucleus pulposus[ALL]) AND (displacement[ALL] OR degeneration[ALL] OR hernia[ALL] ))

AND

(약침[ALL] OR 봉독[ALL] OR Pharmacoacupuncture[ALL] OR Bee venom[ALL])

② **KMbase**

((([ALL=disc] OR [ALL=nucleus pulposus]) AND ([AHLL=displacement] OR [ALL=degeneration] OR [ALL=hernia*])) OR [ALL=추간판 탈출증] OR [ALL=디스크 탈출증]) AND ([ALL=약침] OR [ALL=봉독] OR [ALL=Pharmacoacupuncture] OR [ALL=Bee venom])

③ **KISS**

(((disc OR nucleus pulposus) AND (displacement OR degeneration OR hernia*)) OR (추간판 탈출증 OR 디스크 탈출증) AND (약침 OR 봉독 OR Pharmacoacupuncture OR Bee venom)

④ **NDSL**

(((disc OR nucleus pulposus) AND (displacement OR degeneration OR hernia*)) OR (추간판 탈출증 OR 디스크 탈출증) AND (약침 OR 봉독 OR Pharmacoacupuncture OR Bee venom)

⑤ **KISTI**

(BI: disc hernia*) OR (BI: nucleus pulposus hernia*) OR (BI: disc displacement) OR (BI: nucleus pulposus displacement) OR (BI: disc degeneration) OR (BI: nucleus pulposus degeneration) OR (BI: 추간판 탈출증) OR (BI: 디스크 탈출증)

AND

(BI:약침) OR (BI:봉독) OR (BI:Pharmacoacupuncture) OR (BI:Bee venom)

⑥ **OASIS**

(((disc OR nucleus pulposus) AND (displacement OR degeneration OR hernia*)) OR (추간판 탈출증 OR 디스크 탈출증) AND (약침 OR 봉독 OR Pharmacoacupuncture OR Bee venom)

**5) Chuna manual therapy**

**(1) English**

① **MEDLINE via Pubmed**

#1 Intervertebral Disc Displacement[Mesh]

#2 Sciatica[Mesh]

#3 Polyradiculopathy[Mesh]

#4 (disc OR discs OR disk OR disks OR nucleus pulposus OR sacroilia* OR Sacroiliac-joint)

#5 (displacement OR degeneration OR hernia* OR protru* OR perfora* OR ruptur* OR degenerat* OR degradat* OR displac* OR prolaps* OR avuls* OR extru*)

#6 #4 AND #5

#7 (nerve root OR nerve roots OR nerve)

#8 (compress* OR entrap* OR inflammat* OR disorder*)

#9 #7 AND #8

#10 (polyradiculopathy OR radiculopath* OR radiculiti* OR sciatic)

#11 {or #1, #2, #3, #6, #9, #10}

#12 (tuina OR chiropractic OR spinal manipulation)

#13 #11 AND #12

② **CENTRAL via Cochrane**

#1 Intervertebral disk displacement[ti,ab,kw]

#2 sciatica[ti,ab,kw]

#3 polyradiculopathy[ti,ab,kw]

#4 (disc OR discs OR disk OR disks OR nucleus pulposus OR sacroilia* OR Sacroiliac-joint)[ti,ab,kw]

#5 (displacement OR degeneration OR hernia* OR protru* OR perfora* OR ruptur* OR degenerat* OR degradat* OR displac* OR prolaps* OR avuls* OR extru* )[ti,ab,kw]

#6 #4 AND #5

#7 (nerve root OR nerve roots OR nerve)[ti,ab,kw]

#8 (compress* OR entrap* OR inflammat* OR disorder*)[ti,ab,kw]

#9 #7 AND #8

#10 (polyradiculopathy OR radiculopath* OR radiculiti* OR sciatic)[ti,ab,kw]

#11 {or #1-#3, #6, #9, #10}

#12 (tuina OR chiropractic OR spinal manipulation)[ti,ab,kw]

#13 #11 AND #12

③ **EMBASE**

#1 'Intervertebral disk displacement'/exp

#2 'sciatica'/exp

#3 'polyradiculopathy'/exp

#4 (disc OR discs OR disk OR disks OR nucleus pulposus OR sacroilia* OR Sacroiliac-joint):ab,ti

#5 (displacement OR degeneration OR hernia* OR protru* OR perfora* OR ruptur* OR degenerat* OR degradat* OR displac* OR prolaps* OR avuls* OR extru* ):ab,ti

#6 #4 AND #5

#7 (nerve root OR nerve roots OR nerve):ab,ti

#8 (compress* OR entrap* OR inflammat* OR disorder*):ab,ti

#9 #7 AND #8

#10 (polyradiculopathy OR radiculopath* OR radiculiti* OR sciatic):ab,ti

#11 {or #1-#3, #6, #9, #10}

#12 (tuina OR chiropractic OR spinal manipulation):ab,ti

#13 #11 AND #12

**(2) Chinse**

① **CNKI**

“HIVD” OR “Herniated Intervertebral disc” OR “HNP” OR “Herniated nucleus pulposus” OR “spinal disc herniation” OR “intervertebral disc herniation” OR “herniated disc” OR “Intervertebral disc displacement”

AND

"tuina" OR “chiropractic” OR "spinal manipulation“

**(3) Japanese**

① **CiNII and J-Stage**

((((((((椎間板 OR 神経根症 OR ディスク) OR (Intervertebral Disc Displacement OR Sciatica) OR Polyradiculopathy) OR ((((disc OR discs OR disk OR disks OR nucleus pulposus OR sacroilia* OR Sacroiliac-joint))) AND ((displacement OR degeneration OR hernia* OR protru* OR perfora* OR ruptur* OR degenerat* OR degradat* OR displac* OR prolaps* OR avuls* OR extru*)))) OR ((((nerve root OR nerve roots OR nerve))) AND ((compress* OR entrap* OR inflammat* OR disorder*)))) OR (((polyradiculopathy OR radiculopath* OR radiculiti* OR sciatic))))

AND (tuina OR chiropractic OR spinal manipulation OR 推拏)

**(4) Korean**

① **KoreaMed**

((disc[ALL] OR nucleus pulposus[ALL]) AND (displacement[ALL] OR degeneration[ALL] OR hernia[ALL] ))

AND

(추나[ALL] OR tuina[ALL] OR chiropractic[ALL] OR spinal manipulation[ALL])

② **KMbase**

((([ALL=disc] OR [ALL=nucleus pulposus]) AND ([AHLL=displacement] OR [ALL=degeneration] OR [ALL=hernia*])) OR [ALL=추간판 탈출증] OR [ALL=디스크 탈출증]) AND

([ALL=추나] OR [ALL=Tuina] OR [ALL=chiropractic] OR [ALL=spinal manipulation])

③ **KISS**

(((disc OR nucleus pulposus) AND (displacement OR degeneration OR hernia*)) OR (추간판 탈출증 OR 디스크 탈출증) AND (추나 OR tuina OR chiropractic OR spinal manipulation)

④ **NDSL**

(((disc OR nucleus pulposus) AND (displacement OR degeneration OR hernia*)) OR (추간판 탈출증 OR 디스크 탈출증) AND (추나 OR tuina OR chiropractic OR spinal manipulation)

⑤ **KISTI**

(BI: disc hernia*) OR (BI: nucleus pulposus hernia*) OR (BI: disc displacement) OR (BI: nucleus pulposus displacement) OR (BI: disc degeneration) OR (BI: nucleus pulposus degeneration) OR (BI: 추간판 탈출증) OR (BI: 디스크 탈출증)

AND

(BI: 추나) OR (BI: tuina) OR (BI: chiropractic) OR (BI: spinal manipulation)

⑥ **OASIS**

(((disc OR nucleus pulposus) AND (displacement OR degeneration OR hernia*)) OR (추간판 탈출증 OR 디스크 탈출증) AND (추나 OR tuina OR chiropractic OR spinal manipulation)

**6) Thread-embedding acupuncture**

**(1) English**

① **MEDLINE via Pubmed**

#1 Intervertebral Disc Displacement[Mesh]

#2 Sciatica[Mesh]

#3 Polyradiculopathy[Mesh]

#4 (disc OR discs OR disk OR disks OR nucleus pulposus OR sacroilia* OR Sacroiliac-joint)

#5 (displacement OR degeneration OR hernia* OR protru* OR perfora* OR ruptur* OR degenerat* OR degradat* OR displac* OR prolaps* OR avuls* OR extru*)

#6 #4 AND #5

#7 (nerve root OR nerve roots OR nerve)

#8 (compress* OR entrap* OR inflammat* OR disorder*)

#9 #7 AND #8

#10 (polyradiculopathy OR radiculopath* OR radiculiti* OR sciatic)

#11 {or #1, #2, #3, #6, #9, #10}

#12 (catgut implantation OR thread implantation OR catgut embedding OR embedding therapy OR needle embedding OR Embedded Needle OR Maesun)

#13 #11 AND #12

② **CENTRAL via Cochrane**

#1 Intervertebral disk displacement[ti,ab,kw]

#2 sciatica[ti,ab,kw]

#3 polyradiculopathy[ti,ab,kw]

#4 (disc OR discs OR disk OR disks OR nucleus pulposus OR sacroilia* OR Sacroiliac-joint)[ti,ab,kw]

#5 (displacement OR degeneration OR hernia* OR protru* OR perfora* OR ruptur* OR degenerat* OR degradat* OR displac* OR prolaps* OR avuls* OR extru* )[ti,ab,kw]

#6 #4 AND #5

#7 (nerve root OR nerve roots OR nerve)[ti,ab,kw]

#8 (compress* OR entrap* OR inflammat* OR disorder*)[ti,ab,kw]

#9 #7 AND #8

#10 (polyradiculopathy OR radiculopath* OR radiculiti* OR sciatic)[ti,ab,kw]

#11 {or #1-#3, #6, #9, #10}

#12 (catgut implantation OR thread implantation OR catgut embedding OR embedding therapy OR needle embedding OR Embedded Needle OR Maesun)[ti,ab,kw]

#13 #11 AND #12

③ **EMBASE**

#1 ‘Intervertebral disk displacement’/exp

#2 ‘sciatica’/exp

#3 ‘polyradiculopathy’/exp

#4 (disc OR discs OR disk OR disks OR nucleus pulposus OR sacroilia* OR Sacroiliac-joint):ab,ti

#5 (displacement OR degeneration OR hernia* OR protru* OR perfora* OR ruptur* OR degenerat* OR degradat* OR displac* OR prolaps* OR avuls* OR extru* ):ab,ti

#6 #4 AND #5

#7 (nerve root OR nerve roots OR nerve):ab,ti

#8 (compress* OR entrap* OR inflammat* OR disorder*):ab,ti

#9 #7 AND #8

#10 (polyradiculopathy OR radiculopath* OR radiculiti* OR sciatic):ab,ti

#11 {or #1-#3, #6, #9, #10}

#12 (catgut implantation OR thread implantation OR catgut embedding OR embedding therapy OR needle embedding OR Embedded Needle OR Maesun):ab,ti

#13 #11 AND #12

**(2) Chinese**

① **CNKI**

“HIVD” OR “Herniated Intervertebral disc” OR “HNP” OR “Herniated nucleus pulposus” OR “spinal disc herniation” OR “intervertebral disc herniation” OR “herniated disc” OR “Intervertebral disc displacement”

AND

"catgut implantation" OR "thread implantation" OR "catgut embedding" OR "embedding therapy" OR "needle embedding" OR "Embedded Needle" OR "Maesun“

**(3) Japanese**

① **CiNII and J-Stage**

((((((((椎間板 OR 神経根症 OR ディスク) OR (Intervertebral Disc Displacement OR Sciatica) OR Polyradiculopathy) OR ((((disc OR discs OR disk OR disks OR nucleus pulposus OR sacroilia* OR Sacroiliac-joint))) AND ((displacement OR degeneration OR hernia* OR protru* OR perfora* OR ruptur* OR degenerat* OR degradat* OR displac* OR prolaps* OR avuls* OR extru*)))) OR ((((nerve root OR nerve roots OR nerve))) AND ((compress* OR entrap* OR inflammat* OR disorder*)))) OR (((polyradiculopathy OR radiculopath* OR radiculiti* OR sciatic))))

AND (catgut implantation" OR "thread implantation OR catgut embedding OR embedding therapy OR needle embedding OR Embedded Needle OR Maesun OR 埋線)

**(4) Korean**

① **KoreaMed**

((disc[ALL] OR nucleus pulposus[ALL]) AND (displacement[ALL] OR degeneration[ALL] OR hernia[ALL] ))

AND

(매선[ALL] OR catgut[ALL] OR thread implantation[ALL] OR embedding therapy[ALL] OR needle embedding[ALL])

**② 한국의학논문데이터베이스 (KMbase)**

((([ALL=disc] OR [ALL=nucleus pulposus]) AND ([AHLL=displacement] OR [ALL=degeneration] OR [ALL=hernia*])) OR [ALL=추간판 탈출증] OR [ALL=디스크 탈출증]) AND ([ALL=매선] OR [ALL=catgut] OR [ALL=thread implantation] OR [ALL=embedding therapy] OR [ALL=needle embedding])

**③ 한국학술정보 (KISS)**

(((disc OR nucleus pulposus) AND (displacement OR degeneration OR hernia*)) OR (추간판 탈출증 OR 디스크 탈출증) AND (매선 OR catgut OR thread implantation OR embedding therapy OR needle embedding)

**④ 과학기술정보통합서비스 (NDSL)**

(((disc OR nucleus pulposus) AND (displacement OR degeneration OR hernia*)) OR (추간판 탈출증 OR 디스크 탈출증) AND (매선 OR catgut OR thread implantation OR embedding therapy OR needle embedding)

**⑤ 과학기술학회마을 (KISTI)**

(BI: disc hernia*) OR (BI: nucleus pulposus hernia*) OR (BI: disc displacement) OR (BI: nucleus pulposus displacement) OR (BI: disc degeneration) OR (BI: nucleus pulposus degeneration) OR (BI: 추간판 탈출증) OR (BI: 디스크 탈출증)

AND

(BI: 매선) OR (BI: catgut) OR (BI: thread implantation) OR (BI: embedding therapy) OR (BI: needle embedding)

**⑥ 전통의학정보포탈 (OASIS)**

(((disc OR nucleus pulposus) AND (displacement OR degeneration OR hernia*)) OR (추간판 탈출증 OR 디스크 탈출증) AND (매선 OR catgut OR thread implantation OR embedding therapy OR needle embedding)

**1) Cupping**

**(1) English**

**① MEDLINE via Pubmed**

#1 Intervertebral Disc Displacement[Mesh]

#2 Sciatica[Mesh]

#3 Polyradiculopathy[Mesh]

#4 (disc OR discs OR disk OR disks OR nucleus pulposus OR sacroilia* OR Sacroiliac-joint)

#5 (displacement OR degeneration OR hernia* OR protru* OR perfora* OR ruptur* OR degenerat* OR degradat* OR displac* OR prolaps* OR avuls* OR extru*)

#6 #4 AND #5

#7 (nerve root OR nerve roots OR nerve)

#8 (compress* OR entrap* OR inflammat* OR disorder*)

#9 #7 AND #8

#10 (polyradiculopathy OR radiculopath* OR radiculiti* OR sciatic)

#11 {or #1, #2, #3, #6, #9, #10}

#12 (Cupping)

#13 #11 AND #12

**② CENTRAL via Cochrane**

#1 Intervertebral disk displacement[ti,ab,kw]

#2 sciatica[ti,ab,kw]

#3 polyradiculopathy[ti,ab,kw]

#4 (disc OR discs OR disk OR disks OR nucleus pulposus OR sacroilia* OR Sacroiliac-joint)[ti,ab,kw]

#5 (displacement OR degeneration OR hernia* OR protru* OR perfora* OR ruptur* OR degenerat* OR degradat* OR displac* OR prolaps* OR avuls* OR extru* )[ti,ab,kw]

#6 #4 AND #5

#7 (nerve root OR nerve roots OR nerve)[ti,ab,kw]

#8 (compress* OR entrap* OR inflammat* OR disorder*)[ti,ab,kw]

#9 #7 AND #8

#10 (polyradiculopathy OR radiculopath* OR radiculiti* OR sciatic)[ti,ab,kw]

#11 {or #1-#3, #6, #9, #10}

#12 (Cupping)[ti,ab,kw]

#13 #11 AND #12

③ **EMBASE**

#1 ‘Intervertebral disk displacement’/exp

#2 ‘sciatica’/exp

#3 ‘polyradiculopathy’/exp

#4 (disc OR discs OR disk OR disks OR nucleus pulposus OR sacroilia* OR Sacroiliac-joint):ab,ti

#5 (displacement OR degeneration OR hernia* OR protru* OR perfora* OR ruptur* OR degenerat* OR degradat* OR displac* OR prolaps* OR avuls* OR extru* ):ab,ti

#6 #4 AND #5

#7 (nerve root OR nerve roots OR nerve):ab,ti

#8 (compress* OR entrap* OR inflammat* OR disorder*):ab,ti

#9 #7 AND #8

#10 (polyradiculopathy OR radiculopath* OR radiculiti* OR sciatic):ab,ti

#11 {or #1-#3, #6, #9, #10}

#12 (Cupping):ab,ti

#13 #11 AND #12

**(2) Chinese**

① **CNKI**

“HIVD” OR “Herniated Intervertebral disc” OR “HNP” OR “Herniated nucleus pulposus” OR “spinal disc herniation” OR “intervertebral disc herniation” OR “herniated disc” OR “Intervertebral disc displacement”

AND

"Cupping“

**(3) Japanese**

① **CiNII and J-Stage**

((((((((椎間板 OR 神経根症 OR ディスク) OR (Intervertebral Disc Displacement OR Sciatica) OR Polyradiculopathy) OR ((((disc OR discs OR disk OR disks OR nucleus pulposus OR sacroilia* OR Sacroiliac-joint))) AND ((displacement OR degeneration OR hernia* OR protru* OR perfora* OR ruptur* OR degenerat*

OR degradat* OR displac* OR prolaps* OR avuls* OR extru*)))) OR ((((nerve root OR nerve roots OR nerve))) AND ((compress* OR entrap* OR inflammat* OR disorder*)))) OR (((polyradiculopathy OR radiculopath* OR radiculiti* OR sciatic))))

AND (Cupping OR 附缸)

**(4) Korean**

① **KoreaMed**

((disc[ALL] OR nucleus pulposus[ALL]) AND (displacement[ALL] OR degeneration[ALL] OR hernia[ALL] ))

AND

(부항[ALL] OR Cupping[ALL])

② **KMbase**

((([ALL=disc] OR [ALL=nucleus pulposus]) AND ([AHLL=displacement] OR [ALL=degeneration] OR [ALL=hernia*])) OR [ALL=추간판 탈출증] OR [ALL=디스크 탈출증]) AND ([ALL=부항] OR [ALL=Cupping])

③ **KISS**

(((disc OR nucleus pulposus) AND (displacement OR degeneration OR hernia*)) OR (추간판 탈출증 OR 디스크 탈출증) AND (부항 OR Cupping)

④ **NDSL**

(((disc OR nucleus pulposus) AND (displacement OR degeneration OR hernia*)) OR (추간판 탈출증 OR 디스크 탈출증) AND (부항 OR Cupping)

⑤ **KISTI**

(BI: disc hernia*) OR (BI: nucleus pulposus hernia*) OR (BI: disc displacement) OR (BI: nucleus pulposus displacement) OR (BI: disc degeneration) OR (BI: nucleus pulposus degeneration) OR (BI: 추간판 탈출증) OR (BI: 디스크 탈출증)

AND

(BI:  부항) OR (BI: Cupping)

⑥ **OASIS**

(((disc OR nucleus pulposus) AND (displacement OR degeneration OR hernia*)) OR (추간판 탈출증 OR 디스크 탈출증) AND (부항 OR Cupping)
