# Supplementary material for: Korean Medicine Clinical Practice Guidelines for Lumbar Herniated Intervertebral Disc in Adults: Based on Grading of Recommendations Assessment, Development and Evaluation (GRADE)
Source: Healthcare (Basel). 2022 Jan 27;10(2):246. doi: 10.3390/healthcare10020246 (PMC8871943; doi:10.3390/healthcare10020246)

Supplementary S2.

The Grading of Recommendations Assessment, Development and Evaluation (GRADE) Information.

Outcome measures (Number of studies, study design)	Risk of bias	Inconsistency	Indirectness	Imprecision	Other consideration
**Acupuncture**					
CQ1. Total clinical effective rate (23, RCTs)[12-35]	Not serious	Not serious	Not serious	Not serious	Not serious
Pain (15, RCTs)[12-26]	Not serious	Serious	Not serious	Not serious	Not serious
Function (4, RCTs)[13,14,16,33]	Not serious	Serious	Not serious	Serious	Not serious
CQ2. Total clinical effective rate (13, RCTs)[20, 36-44]	Not serious	Serious	Not serious	Not serious	Not serious
Pain (5, RCTs)[36, 42-45]	Not serious	Serious	Not serious	Not serious	Not serious
Function (ODI) (2, RCTs)[36, 43]	Not serious	Not serious	Not serious	Serious	Not serious
Function (JOA) (3, RCTs)[44,46,47]	Not serious	Serious	Not serious	Serious	Not serious
CQ3.Total clinical effective rate (7, RCTs)[22, 53-57]	Not serious	Not serious	Not serious	Not serious	Not serious
Pain (6, RCTs)[52-56]	Not serious	Serious	Not serious	Not serious	Not serious
Function (ODI) (2, RCTs)[52, 55]	Not serious	Not serious	Not serious	Serious	Not serious
CQ4.Total clinical effective rate (7, RCTs)[59-65]	Not serious	Serious	Not serious	Not serious	Not serious

Pain (1, RCTs)[58]	Serious	Not serious	Not serious	Very serious	Not serious
**Moxibustion**					
CQ5.Total clinical effective rate (1, RCTs)[66]	Not serious	Not serious	Not serious	Very serious	Not serious
CQ6.Total clinical effective rate (5, RCTs)[47, 57, 69-71]	Not serious	Not serious	Not serious	Not serious	Not serious
Pain (1, RCTs)[69]	Not serious	Not serious	Not serious	Serious	Not serious
Function (JOA) (4, RCTs)[47, 69-71]	Not serious	Serious	Not serious	Not serious	Not serious
CQ7.Total clinical effective rate (2, RCTs)[74,75]	Serious	Not serious	Not serious	Serious	Not serious
Function (m-JOA) (2, RCTs)[72,73]	Not serious	Not serious	Not serious	Serious	Not serious
**Herbal medicine**					
CQ8. Total clinical effective rate (5, RCTs)[76, 79-82]	Not serious	Not serious	Not serious	Not serious	Not serious
Pain (3, RCTs)[76-78]	Not serious	Serious	Not serious	Serious	Not serious
Function (ODI) (3, RCTs)[76-78]	Not serious	Serious	Not serious	Serious	Not serious
Function (JOA) (1, RCTs)[79]	Not serious	Not serious	Not serious	Serious	Not serious
CQ9. Total clinical effective rate (21, RCTs)[40,41,83,85-100,102-103]	Not serious	Not serious	Not serious	Not serious	Not serious
Pain (10, RCTs)[83,84,94,97-103]	Not serious	Serious	Not serious	Not serious	Not serious
Function (ODI) (1, RCTs)[83]	Serious	Not serious	Not serious	Serious	Not serious

Function (JOA) (4, RCTs)[85,97,98,101]	Not serious	Serious	Not serious	Not serious	Not serious
**Pharmacopuncture**					
CQ10. Total clinical effective rate (5, RCTs)[76, 105, 107, 109]	Not serious	Not serious	Not serious	Serious	Not serious
Pain (8, RCTs)[97, 104-109]	Not serious	Not serious	Not serious	Not serious	Not serious
Function (ODI) (1, RCTs)[110]	Not serious	Not serious	Not serious	Serious	Not serious
Function (ODIc) (3, RCTs)[104, 106, 109]	Not serious	Not serious	Not serious	Serious	Not serious
Function (JOA) (1, RCTs)[97]	Not serious	Not serious	Not serious	Serious	Not serious
**Chuna manual therapy**					
CQ11. Total clinical effective rate (10, RCTs)[111-120]	Not serious	Not serious	Not serious	Not serious	Not serious
Pain (5, RCTs)[111-115]	Not serious	Not serious	Not serious	Not serious	Not serious
Function (ODI) (1, RCTs)[113]	Not serious	Not serious	Not serious	Serious	Not serious
Function (JOA) (1, RCTs)[116]	Not serious	Not serious	Not serious	Serious	Not serious
CQ12. Total clinical effective rate (29, RCTs)[36, 39, 89, 99, 121, 124-147]	Not serious	Serious	Not serious	Not serious	Not serious
Pain (8, RCTs)[36, 99, 121-126]	Not serious	Serious	Not serious	Not serious	Not serious
Function (ODI) (2, RCTs)[36, 124]	Not serious	Serious	Not serious	Serious	Not serious
Function (JOA) (4, RCTs)[46, 124, 127, 128]	Not serious	Serious	Not serious	Not serious	Not serious

**Thread-embedding acupuncture**					
CQ13. Total clinical effective rate (12, RCTs)[148-159]	Serious	Not serious	Not serious	Not serious	Not serious
Pain (4, RCTs)[148-151]	Serious	Serious	Not serious	Serious	Not serious
Function (ODI) (1, RCTs)[151]	Not serious	Not serious	Not serious	Serious	Not serious
Function (JOA) (1, RCTs)[148]	Not serious	Not serious	Not serious	Serious	Not serious
CQ14. Total clinical effective rate (6, RCTs)[160, 162-166]	Not serious	Serious	Not serious	Not serious	Not serious
Pain (2, RCTs)[160, 161]	Not serious	Serious	Not serious	Serious	Not serious
Function (ODI) (2, RCTs)[160, 161]	Not serious	Serious	Not serious	Serious	Not serious
Function (JOA) (1, RCTs)[160]	Not serious	Not serious	Not serious	Serious	Not serious
**Cupping**					
CQ15. Total clinical effective rate (5, RCTs)[167-171]	Not serious	Not serious	Not serious	Not serious	Not serious
Pain (1, RCTs)[167]	Not serious	Not serious	Not serious	Very serious	Not serious

PRISMA flow chart

CQ1. Acupuncture vs. active control treatment

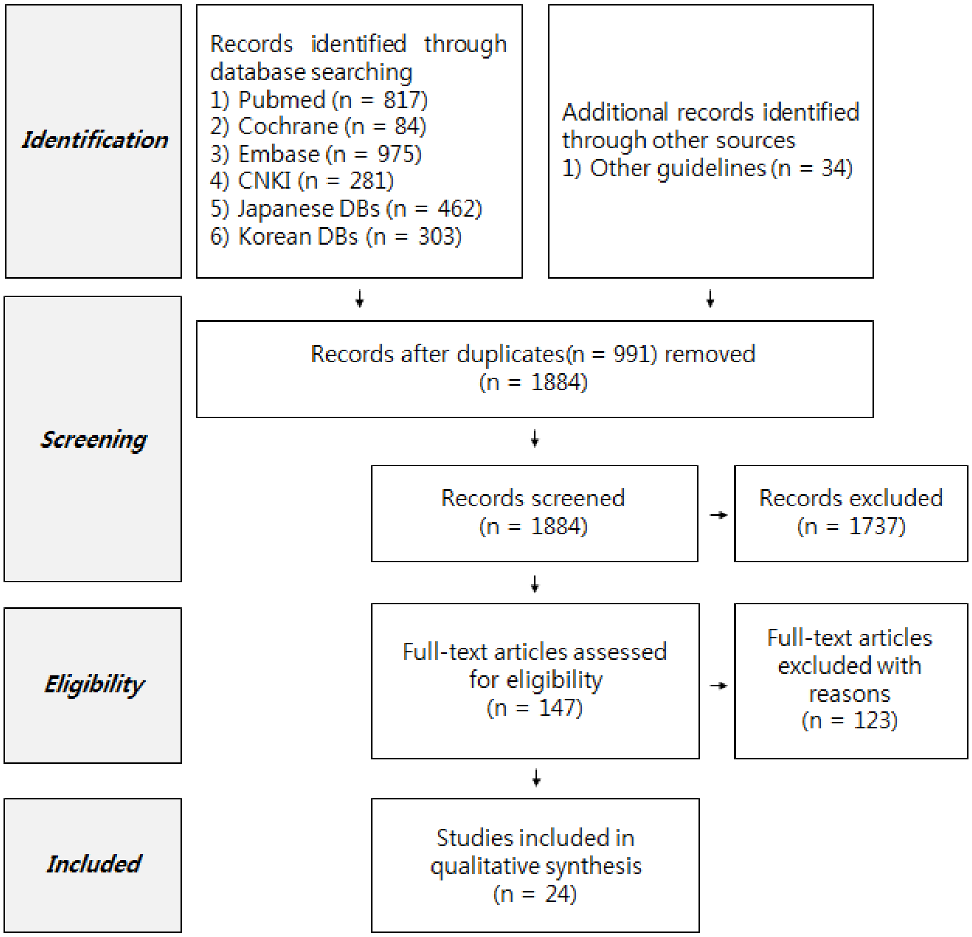

CQ2. Acupuncture + usual conventional therapy vs. acupuncture

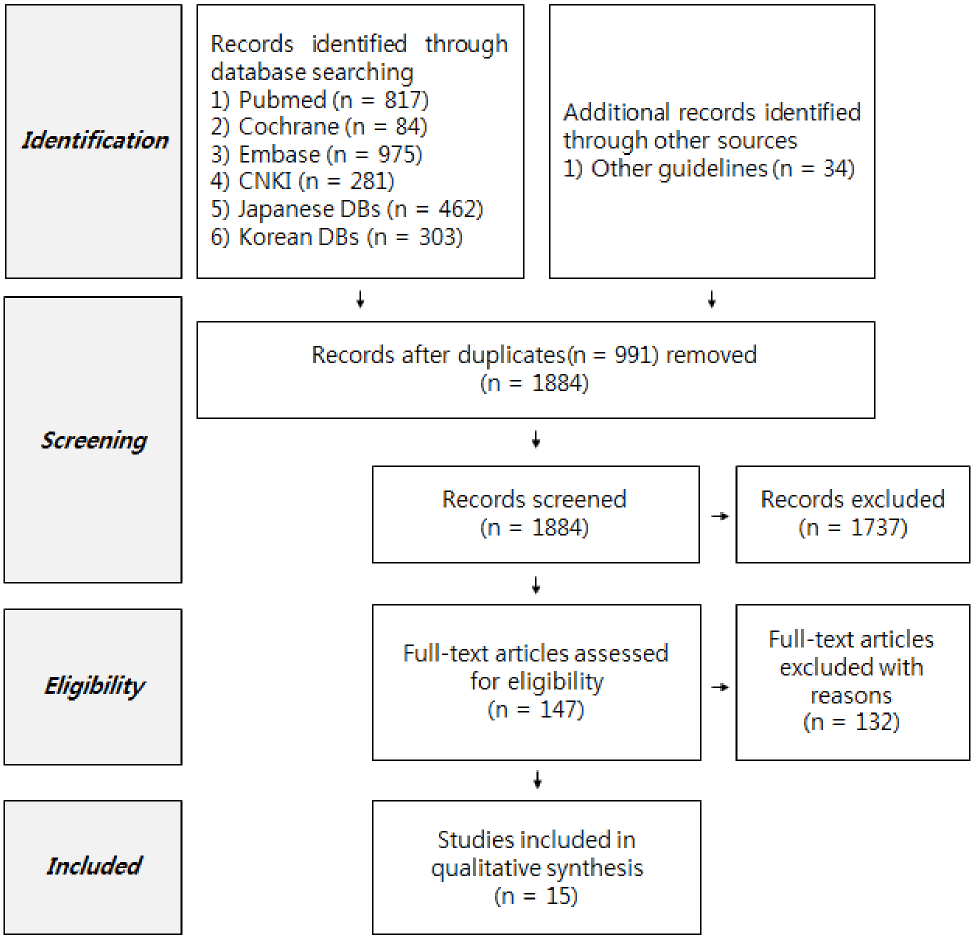

CQ3. Electroacupuncture, fire needling or warm needling vs. acupuncture

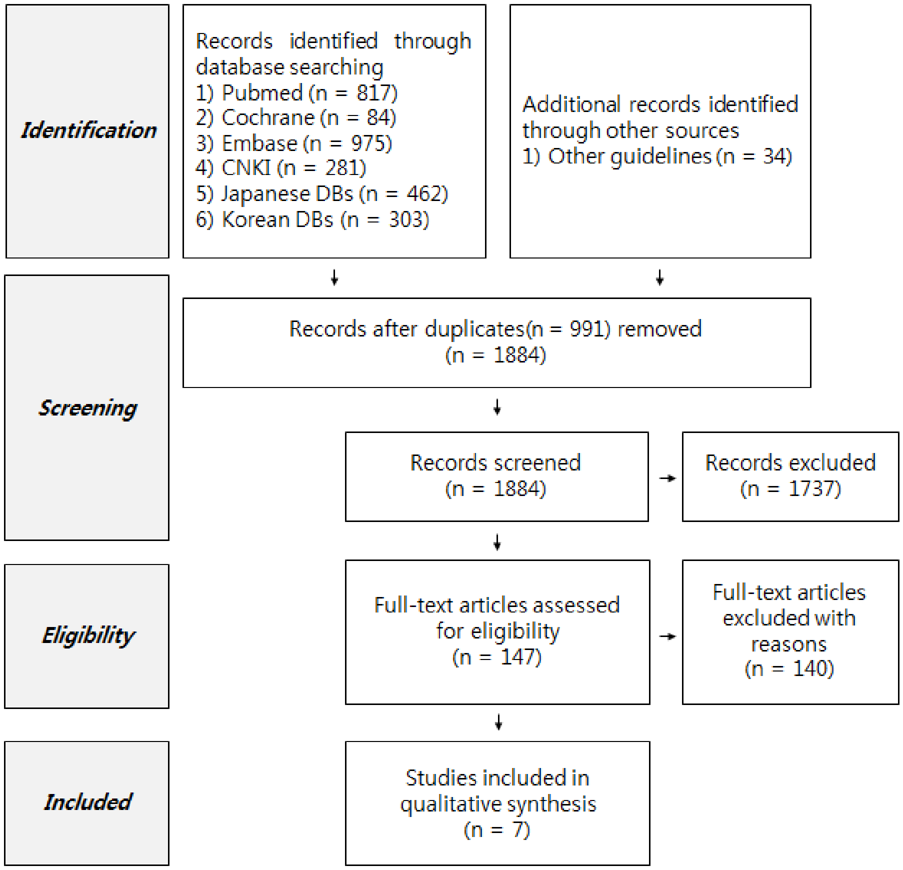

CQ4. Deep insertion acupuncture vs. superficial insertion acupuncture

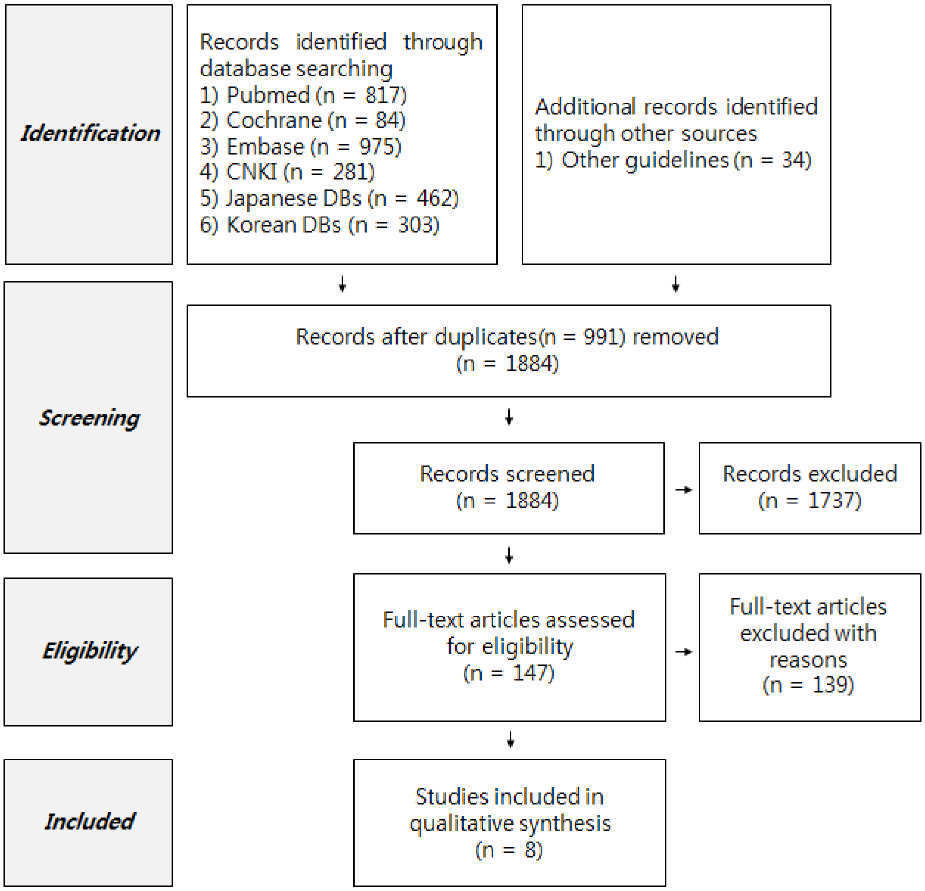

CQ5. Moxibustion vs. active control group

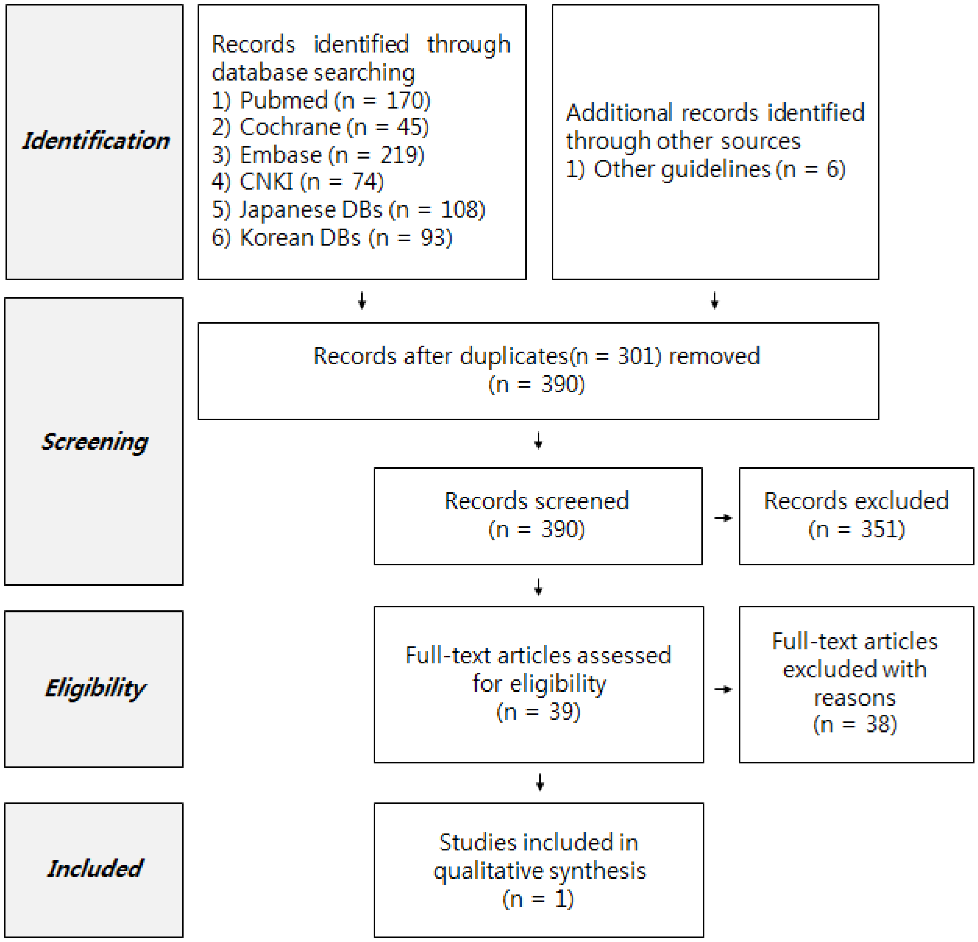

CQ6. Moxibustion + usual conventional therapy vs. moxibustion

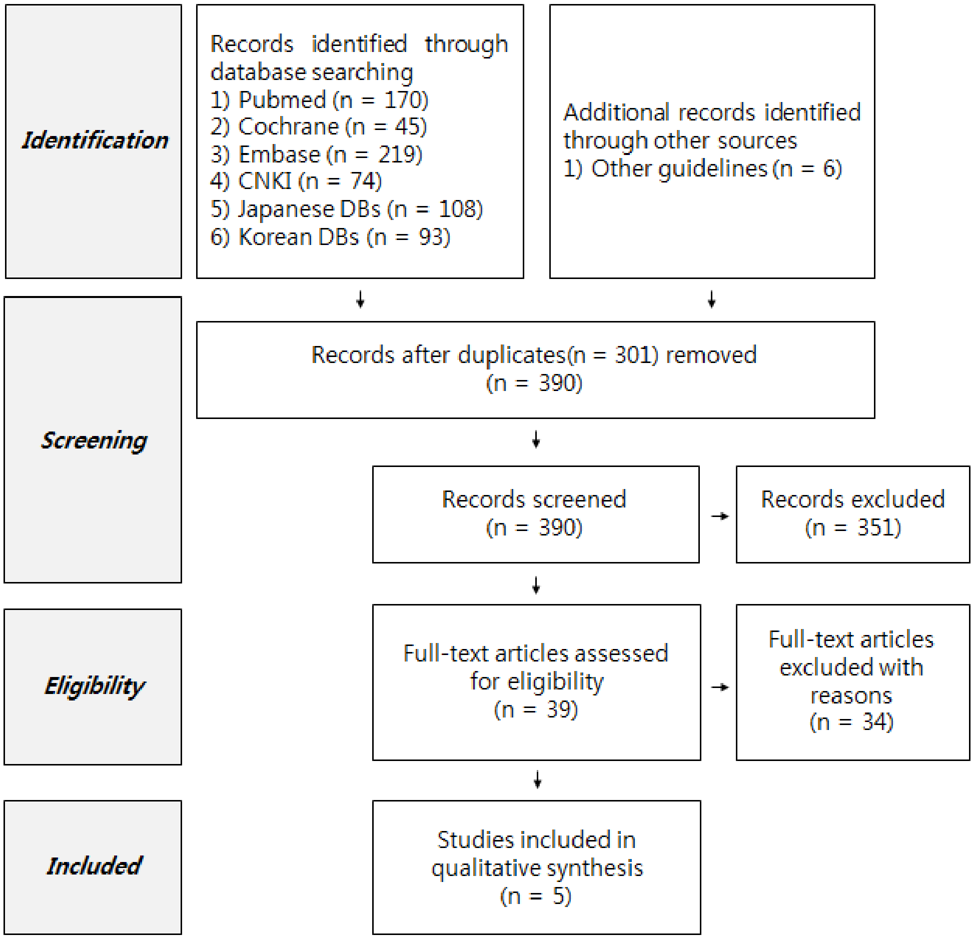

CQ7. Moxibustion causing deqi sensation vs. moxibustion not causing deqi sensation

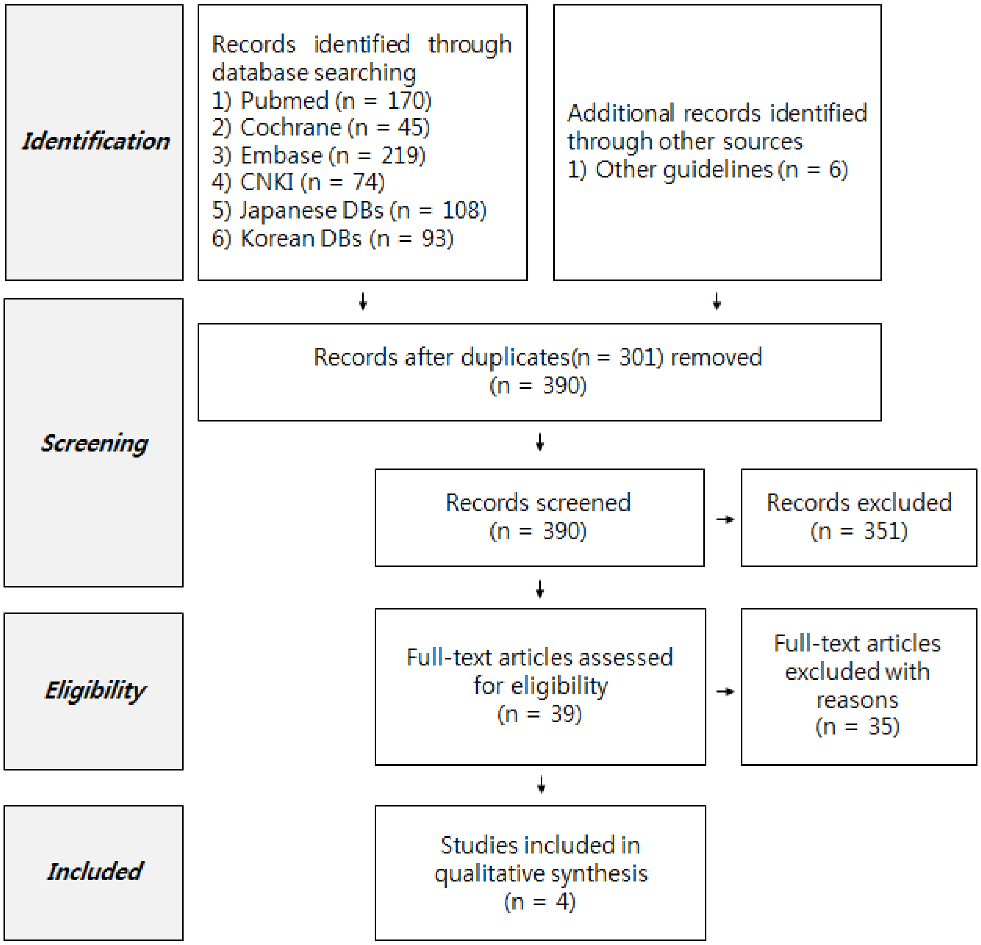

CQ8. Herbal medicine vs. active control treatment

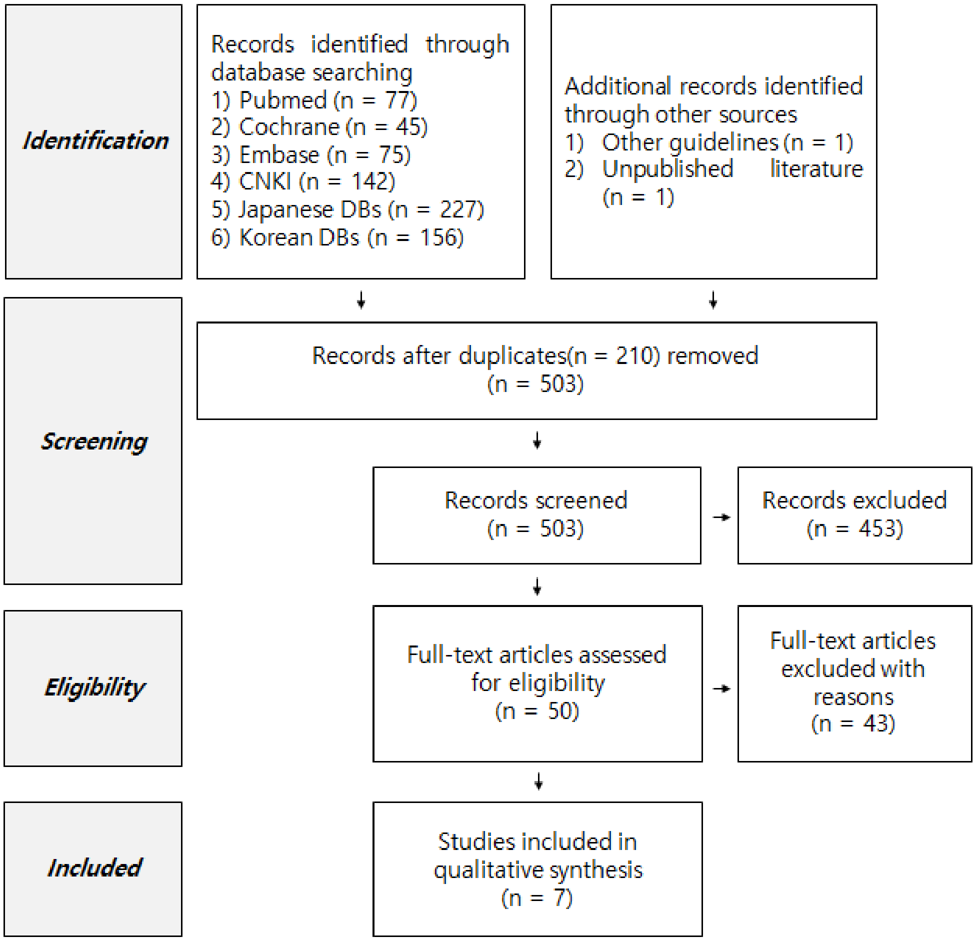

CQ9. Herbal medicine + usual conventional therapy vs. herbal medicine

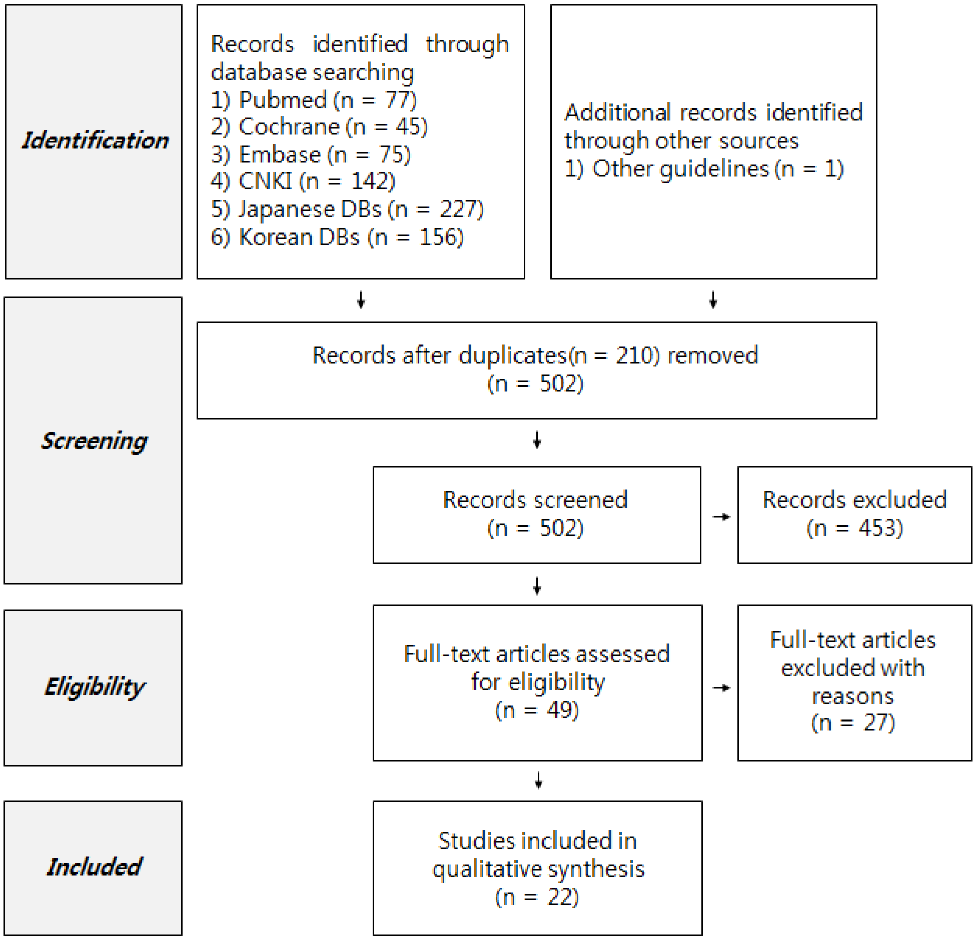

CQ10. Pharmacopuncture + usual conventional therapy vs. pharmacopuncture

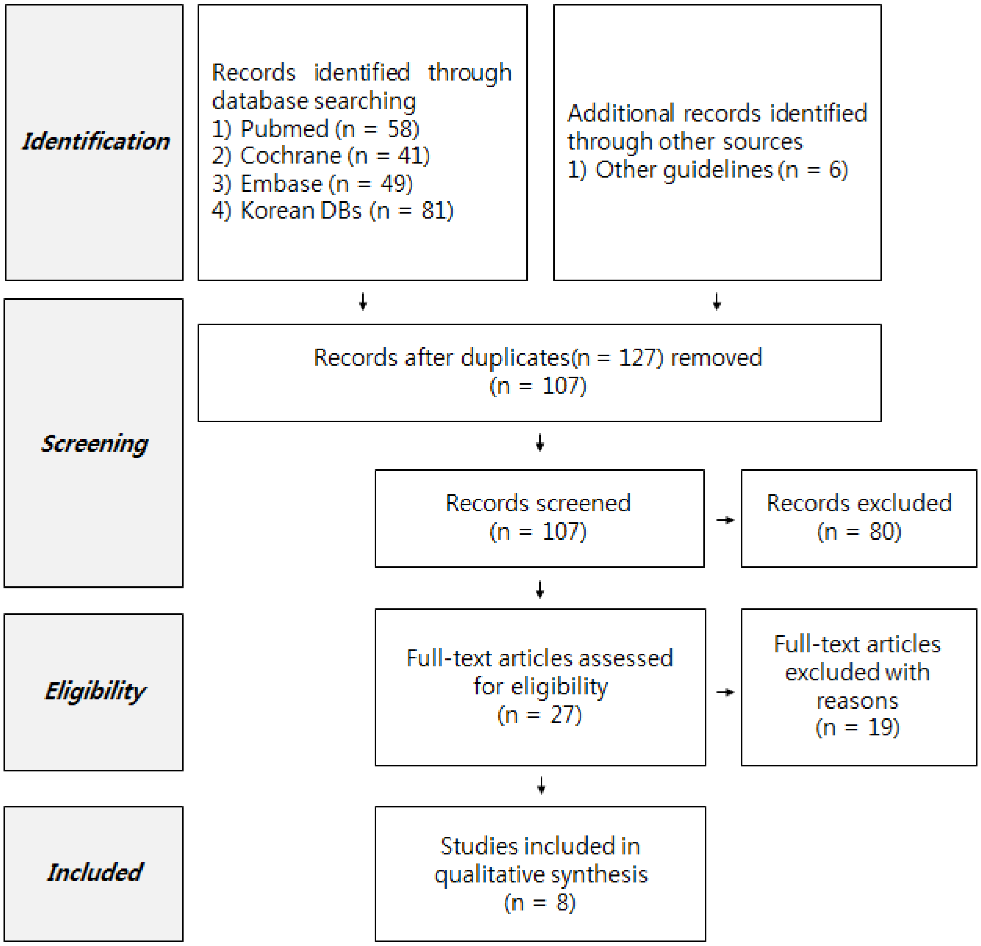

CQ11. Chuna manual therapy vs, active control treatment

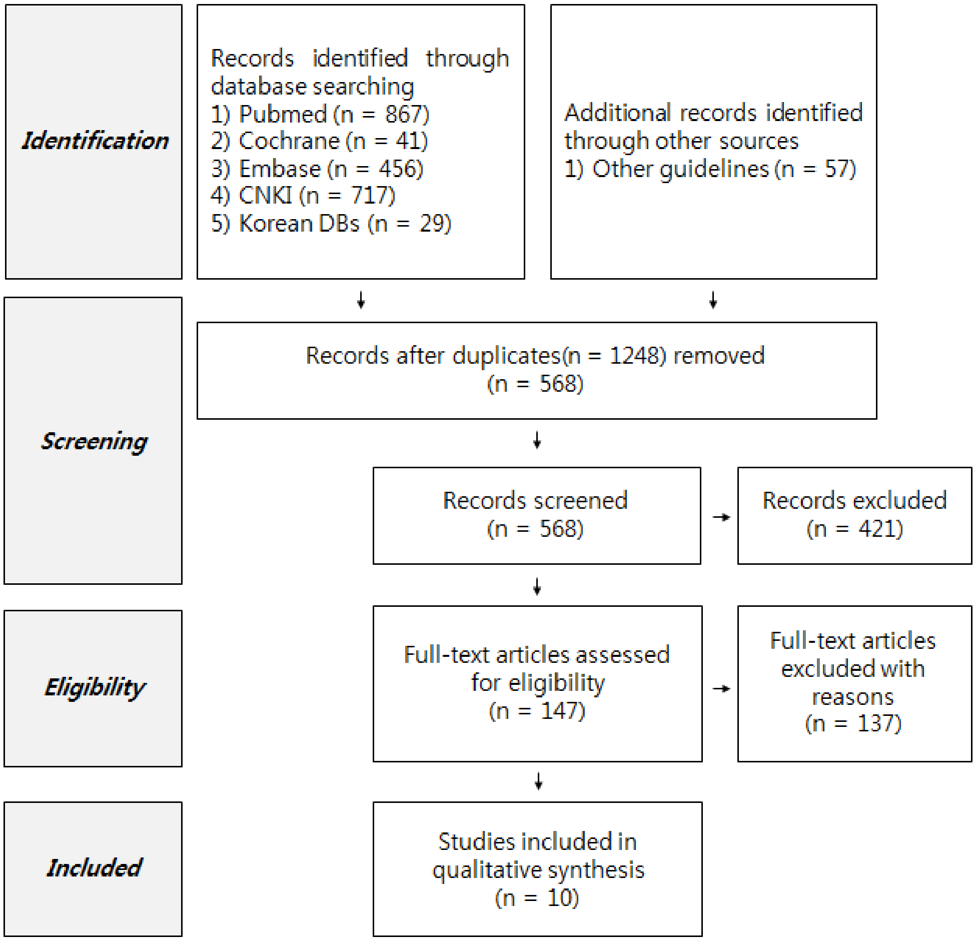

CQ12. Chuna manual therapy + usual conventional therapy vs. usual conventional therapy

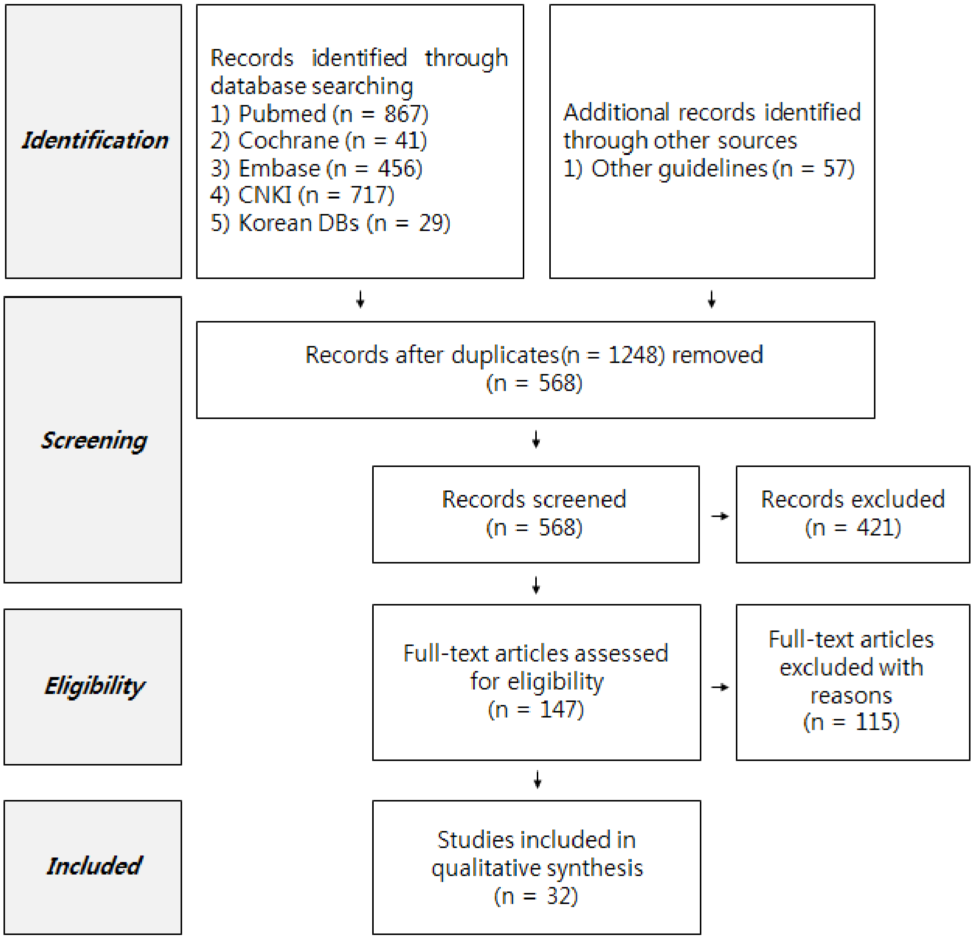

CQ13. TEA vs. active control treatment

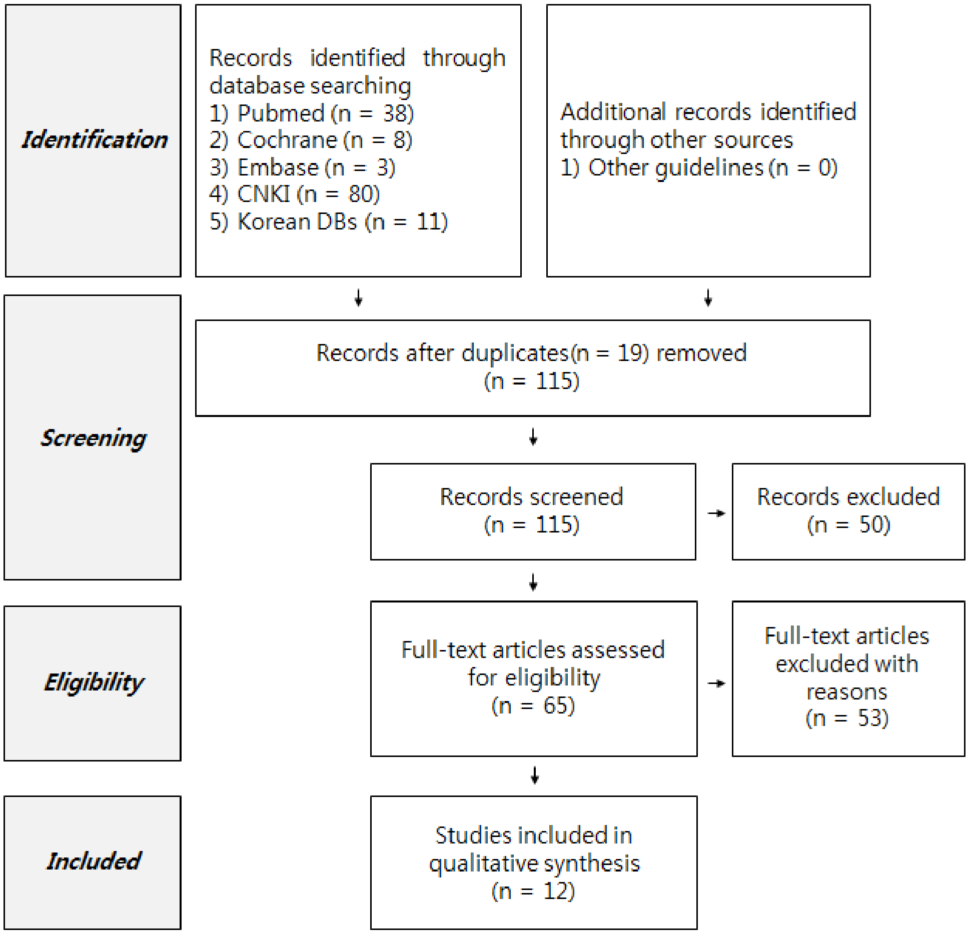

CQ14. TEA + usual conventional therapy vs. TEA

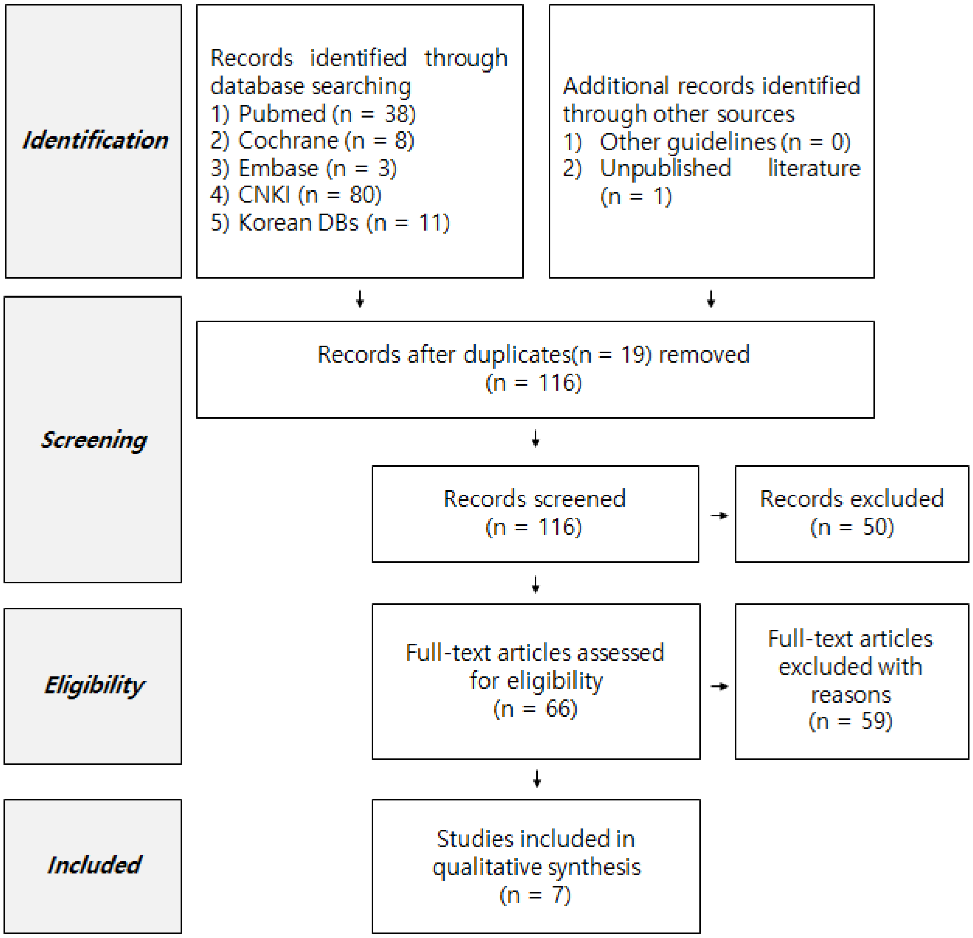

CQ15. Cupping + usual conventional therapy vs. usual conventional therapy

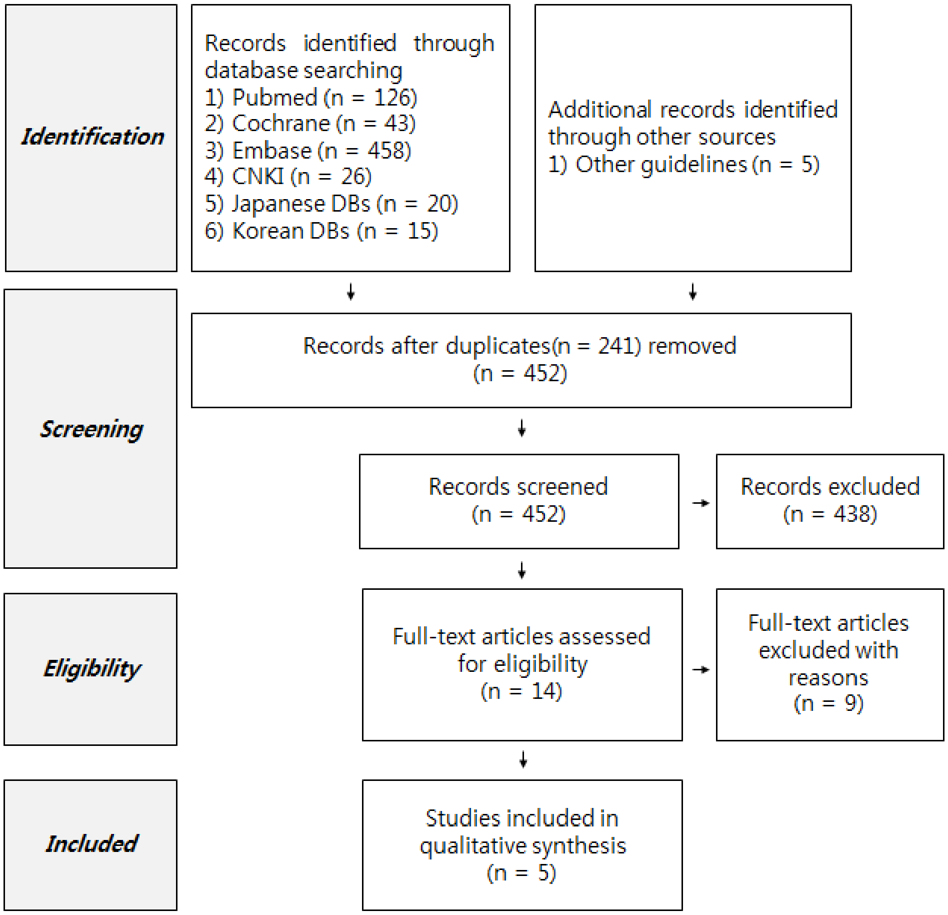

Supplement: Supplementary file 1 [file healthcare-10-00246-s001.zip › Supplementary S2.pdf]
