# Supplementary material for: Korean Medicine Clinical Practice Guidelines for Lumbar Herniated Intervertebral Disc in Adults: Based on Grading of Recommendations Assessment, Development and Evaluation (GRADE)
Source: Healthcare (Basel). 2022 Jan 27;10(2):246. doi: 10.3390/healthcare10020246 (PMC8871943; doi:10.3390/healthcare10020246)
Supplement: Supplementary file 1 [file healthcare-10-00246-s001.zip › Supplementary S3.pdf]

Supplementary S3

Details of Recommendation

Recommendation number	Recommendation content		Recommendation level / evidence Level
**1) Acupuncture**			
**Acupuncture alone treatment**			
**R1**	**Acupuncture alone treatment is recommended for improving overall symptoms of the lumbar herniated intervertebral disc in adult**		**A / High**
	**Clinical considerations**<br>EX-B2 and acupoints of Governor Vessel(GV) and Bladder Meridian(BL) at the lumbar region may be primarily considered, and acupoints of BL and Gallbladder Meridian (GB) at the hip and lower extremities may be considered additionally depending on the location of the radiation pain.<br>The retention time of acupuncture or stimulation time of electro-acupuncture is generally appropriate for 20-30 minutes.		
**Combination treatment**			
**R2**	**A combination of acupuncture with usual care is recommended for improving overall symptoms of the lumbar herniated intervertebral disc in adults**		**A / Moderate**
	**Clinical considerations**<br>A combination of acupuncture with Korean medical treatment such as moxibustion, herbal medicine, or chuna or Western medical treatment such as oral medication or injection may be considered to improve the effectiveness.		
**Treatment method**			

**R3**	**Additional heat or electric stimulation on acupuncture is recommended for improving overall symptoms of the lumbar herniated intervertebral disc in adults**	**A / High**
**R4**	**Deep insertion acupuncture technique should be considered for improving overall symptoms of the lumbar herniated intervertebral disc in adults**	**B / Moderate**
	**Clinical considerations**<br>Deep insertion acupuncture technique is mainly applicable to EX-B2, and an acupuncture needle may be inserted to a depth of 45-90mm or bone-contact depth depending on the patient's physique for safety.	
**2) Moxibustion**		
**Moxibustion alone treatment**		
**R5**	**Based on the expert group consensus, moxibustion alone treatment is recommended for improving overall symptoms of the lumbar herniated intervertebral disc in adult**	**GPP / Very low**
**Combination treatment**		
**R6**	**A combination of moxibustion with acupuncture or chuna is recommended for improving overall symptoms of the lumbar herniated intervertebral disc in adults**	**A / High**
	**Clinical considerations**<br>BL23, GB30, BL40, BL25 and ashi points can be considered when selecting acupoints for moxibustion.	
**Treatment method**		
**R7**	**Moxibustion that elicits Deqi sensation may be considered for improving overall symptoms of**	**C / Low**

	**the lumbar herniated intervertebral disc in adults**	
**3) Herbal medicine**		
**Herbal medicine alone treatment**		
**R8**	**Herbal medicine alone treatment should be considered for improving overall symptoms of the lumbar herniated intervertebral disc in adult**	**B / Moderate**
	**Clinical considerations**<br>Dokwhalgisaeng-tang which can be modified by pattern identification may be prescribed in herbal medicine alone treatment for the lumbar herniated intervertebral disc.	
**Combination treatment**		
**R9**	**A combination of herbal medicine with usual care is recommended for improving overall symptoms of the lumbar herniated intervertebral disc in adults**	**A / High**
	**Clinical considerations**<br>A combination of herbal medicine with Korean medical treatment such as acupuncture, pharmacopuncture, or chuna, or western medical treatment such as oral medications or injection may be considered to improve the effectiveness.<br>Dokwhalgisaeng-tang which can be modified by pattern identification may be prescribed in herbal medicine combination treatment for the lumbar herniated intervertebral disc.	
**4) Pharmacopuncture**		
**Combination treatment**		
**R10**	**A combination of pharmacopuncture with usual care should be considered for improving overall**	**B / Moderate**

<table>
<tr><td></td><td>symptoms of the lumbar herniated intervertebral disc in adults</td><td></td></tr>
<tr><td></td><td colspan="2">Clinical considerations
A combination of pharmacopuncture with acupuncture, chuna or integrative Korean medical treatment may be considered to improve the effectiveness.
Bee venom Pharmacopuncture may be considered when selecting pharmacopuncture for the lumbar herniated intervertebral disc, and the dose or concentration of bee venom should be gradually increased after confirming a patient's adverse reaction.
EX-B2, acupoints of Governor Vessel(GV), and ashi points may be considered for pharmacopuncture treatment.</td></tr>
<tr><td colspan="3">5) Chuna</td></tr>
<tr><td colspan="3">Chuna alone treatment</td></tr>
<tr><td>R11</td><td>Chuna alone treatment is recommended for improving overall symptoms of the lumbar herniated intervertebral disc in adult</td><td>A / High</td></tr>
<tr><td colspan="3">Combination treatment</td></tr>
<tr><td rowspan="2">R12</td><td>A combination of chuna with usual care is recommended for improving overall symptoms of the lumbar herniated intervertebral disc in adults</td><td>A / Moderate</td></tr>
<tr><td colspan="2">Clinical considerations
A combination of chuna with Korean medical treatment such as acupuncture, herbal medicine, or traction, or western medical treatment such as injection may be considered to improve the effectiveness.</td></tr>
<tr><td colspan="3">6) Thread-embedding acupuncture</td></tr>
<tr><td colspan="3">Thread-embedding acupuncture alone treatment</td></tr>
<tr><td>R13</td><td>Thread-embedding acupuncture should be</td><td>B / Moderate</td></tr>
</table>

	**considered for improving overall symptoms of the lumbar herniated intervertebral disc in adult**	
	**Clinical considerations**<br>EX-B2, acupoints of Governor Vessel (GV) and Bladder Meridian (BL), and ashi points at the lumbar region may be primarily considered, and acupoints of BL and Gallbladder Meridian (GB) at the hip and lower extremities may be considered additionally depending on the location of the radiation pain.<br>When repeating thread-embedding acupuncture treatment, it may be considered at intervals of 1-2 weeks.	
**Combination treatment**		
**R14**	**A combination of thread-embedding acupuncture with usual care should be considered for improving overall symptoms of the lumbar herniated intervertebral disc in adults**	**B / Moderate**
	**Clinical considerations**<br>A combination of thread-embedding acupuncture with electro-acupuncture on the same or similar acupoints may be considered to improve the effectiveness.	
**7) Cupping treatment**		
**Combination treatment**		
**R15**	**A combination of cupping treatment with usual care should be considered for improving overall symptoms of the lumbar herniated intervertebral disc in adults**	**B / Moderate**
	**Clinical considerations**<br>Wet cupping technique may be considered for the treatment of the lumbar herniated intervertebral disc, and it may be combined with	

	acupuncture to improve the effectiveness.

Diagnostic algorithm

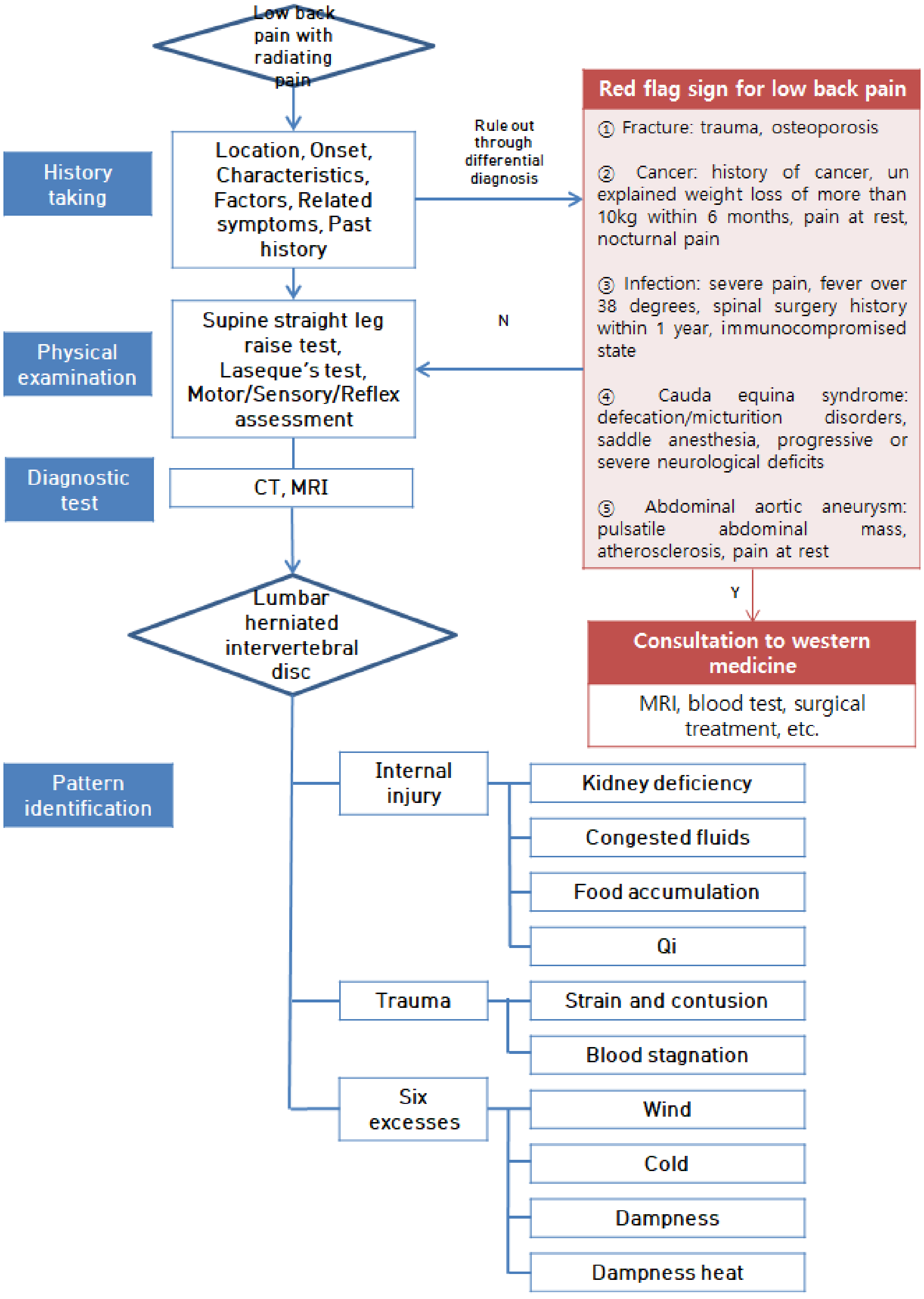

Treatment algorithm

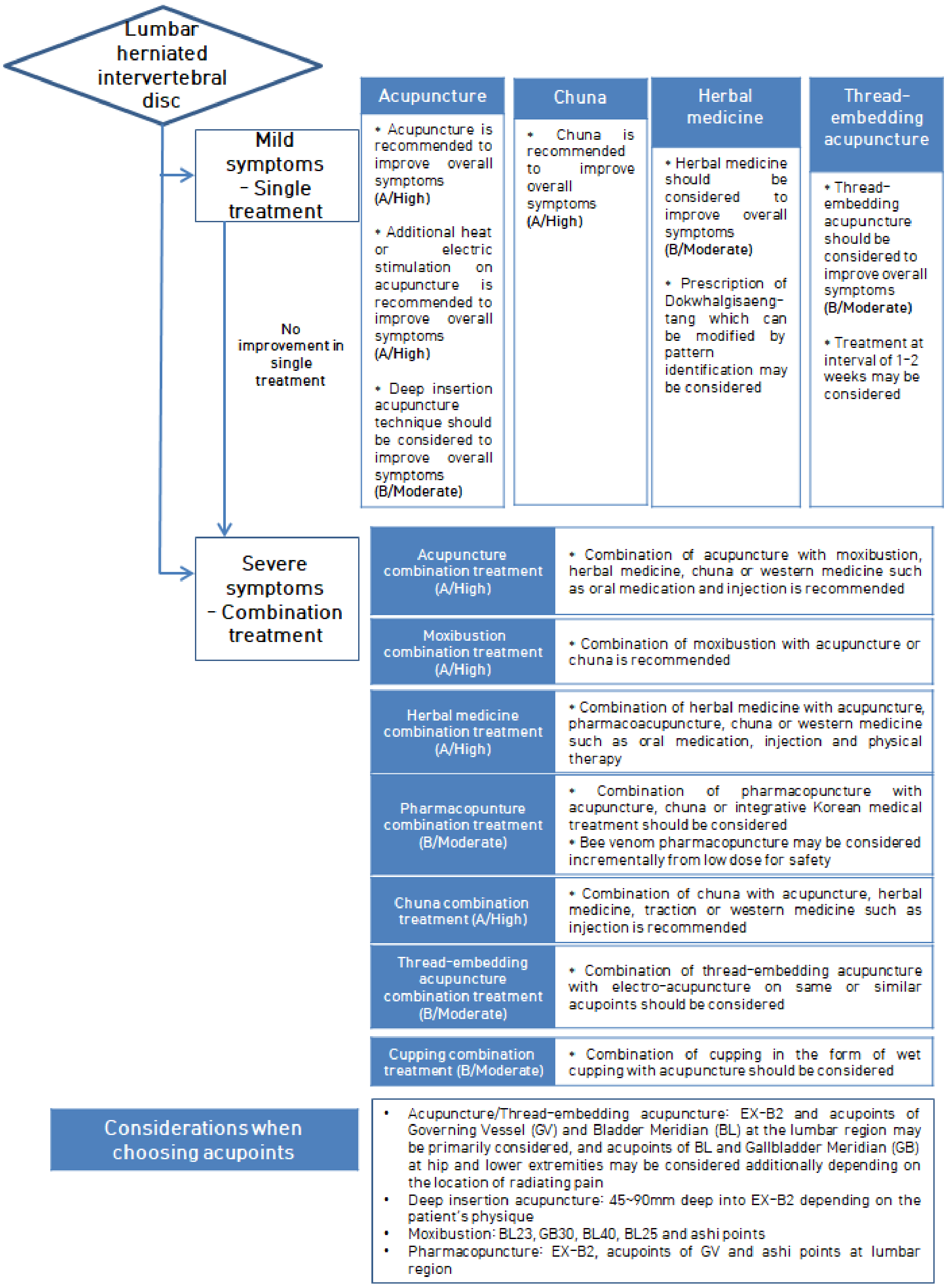

Lumbar herniated intervertebral disc
Mild symptoms - Single treatment
No improvement in single treatment
Severe symptoms - Combination treatment
Acupuncture
• Acupuncture is recommended to improve overall symptoms (A/High)
• Additional heat or electric stimulation on acupuncture is recommended to improve overall symptoms (A/High)
• Deep insertion acupuncture technique should be considered to improve overall symptoms (B/Moderate)
Chuna
• Chuna is recommended to improve overall symptoms (A/High)
Herbal medicine
• Herbal medicine should be considered to improve overall symptoms (B/Moderate)
• Prescription of Dokwhalgisaeng-tang which can be modified by pattern identification may be considered
Thread-embedding acupuncture
• Thread-embedding acupuncture should be considered to improve overall symptoms (B/Moderate)
• Treatment at interval of 1-2 weeks may be considered
Acupuncture combination treatment (A/High)
• Combination of acupuncture with moxibustion, herbal medicine, chuna or western medicine such as oral medication and injection is recommended
Moxibustion combination treatment (A/High)
• Combination of moxibustion with acupuncture or chuna is recommended
Herbal medicine combination treatment (A/High)
• Combination of herbal medicine with acupuncture, pharmacoacupuncture, chuna or western medicine such as oral medication, injection and physical therapy
Pharmacopunture combination treatment (B/Moderate)
• Combination of pharmacopuncture with acupuncture, chuna or integrative Korean medical treatment should be considered
• Bee venom pharmacopuncture may be considered incrementally from low dose for safety
Chuna combination treatment (A/High)
• Combination of chuna with acupuncture, herbal medicine, traction or western medicine such as injection is recommended
Thread-embedding acupuncture combination treatment (B/Moderate)
• Combination of thread-embedding acupuncture with electro-acupuncture on same or similar acupoints should be considered
Cupping combination treatment (B/Moderate)
• Combination of cupping in the form of wet cupping with acupuncture should be considered
Considerations when choosing acupoints
• Acupuncture/Thread-embedding acupuncture: EX-B2 and acupoints of Governing Vessel (GV) and Bladder Meridian (BL) at the lumbar region may be primarily considered, and acupoints of BL and Gallbladder Meridian (GB) at hip and lower extremities may be considered additionally depending on the location of radiating pain
• Deep insertion acupuncture: 45~90mm deep into EX-B2 depending on the patient's physique
• Moxibustion: BL23, GB30, BL40, BL25 and ashi points
• Pharmacopuncture: EX-B2, acupoints of GV and ashi points at lumbar region

Details of Clinical Findings

Outcome measures	Numbar of patients		Effect		Comments
	Intervention group (n)	Comparison group (n)	Relative effect (95% CI, p value)	Number of total patients (Number of studies)	
**Acupuncture**					
CQ1. Total clinical effective rate (23, RCTs) [12-35]	Acupuncture (1,294)	UCT (1,149)	RR 1.18 (1.12,1.25, p<0.00001)	2,443 (23)	The higher score, the more effect
Pain (15, RCTs) [12-26]	Acupuncture (684)	UCT (605)	MD -1.86 (-1.91,-1.81, p<0.00001)	1,289 (15)	The lower score, the lower pain intensity
Function (JOA) (4, RCTs) [13,14,16,33]	Acupuncture (144)	UCT (133)	MD 4.48 (3.39, 5.03, p<0.00001)	277 (4)	The higher score, the more improvement
CQ2. Total clinical effective rate (13, RCTs)[20, 36-44]	Acupuncture + UCT (693)	UCT (632)	RR 1.21 (1.16, 1.28, p<0.00001)	1,326 (13)	The higher score, the more effect

Pain (5, RCTs)[36, 42-45]	Acupuncture + UCT (212)	UCT (212)	MD -1.03 (-1.16,-0.90, p<0.00001)	424 (5)	The lower score, the lower pain intensity
Function (ODI) (2, RCTs) [36, 43]	Acupuncture + UCT (90)	UCT (90)	MD -3.27 (-3.86, -2.68, p<0.00001)	180 (2)	The lower score, the more improvement
Function (JOA) (3, RCTs) [44,46,47]	Acupuncture + UCT (130)	UCT (100)	MD 4.00 (3.48, 4.52, p<0.00001)	200 (3)	The higher score, the more improvement
CQ3. Total clinical effective rate (7, RCTs) [22, 53-57]	Electric acupuncture or fire needling or warm needling (396)	Acupuncture (389)	RR 1.16 (1.09, 1.23, p<0.00001)	785 (7)	The higher score, the more effect
Pain (6, RCTs) [52-56]	Electric acupuncture or fire needling or warm needling (264)	Acupuncture (257)	MD -0.58 (-0.76,-0.39, p<0.00001)	521 (6)	The lower score, the lower pain intensity
Function (ODI) (2, RCTs) [52, 55]	Electric acupuncture or	Acupuncture (67)	MD -0.71 (-1.29,-0.13, p=0.02)	135 (2)	The lower score, the more

	fire needling or warm needling (68)				improvement
CQ4. Total clinical effective rate (7, RCTs) [59-65]	Deep-injection acupuncture (627)	Superficial-injection acupuncture (538)	RR 1.31 (1.23, 1.39, p<0.00001)	1,165 (7)	The higher score, the more effect
Pain (1, RCTs) [58]	Deep-injection acupuncture (10)	Superficial-injection acupuncture (9)	MD -1.66 (-3.97,0.65, p=0.16)	19 (1)	The lower score, the lower pain intensity
**Moxibustion**					
CQ5. Total clinical effective rate (1, RCTs) [66]	Moxibustion (60)	UCT (60)	RR 1.15 (0.97, 1.36, P=0.10)	120 (1)	The higher score, the more effect
CQ6. Total clinical effective rate (5, RCTs) [47, 57, 69-71]	Moxibustion + acupuncture or Moxibustion + Chuna manual therapy (209)	Acupuncture or Chuna manual therapy (179)	RR 1.22 (1.12, 1.32, P=0.04)	388 (5)	The higher score, the more effect

Pain (1, RCTs) [69]	Moxibustion + acupuncture or Moxibustion + Chuna manual therapy (47)	Acupuncture or Chuna manual therapy (48)	MD -1.40 (-1.85,-0.95, p<0.00001)	95 (1)	The lower score, the lower pain intensity
Function (JOA) (4, RCTs) [47, 69-71]	Moxibustion + acupuncture or Moxibustion + Chuna manual therapy (170)	Acupuncture or Chuna manual therapy (140)	MD 4.10 (3.42, 4.77, p<0.00001)	310 (4)	The higher score, the more improvement
CQ7. Total clinical effective rate (2, RCTs) [74,75]	Moxibustion with deqi sensation (120)	Moxibustion without deqi sensation (120)	RR 1.19 (1.06, 1.33, P=0.004)	240 (2)	The higher score, the more effect
Function (m-JOA) (2, RCTs) [72,73]	Moxibustion with deqi sensation (94)	Moxibustion without deqi sensation (94)	MD -2.66 (-4.02,- 1.30, p<0.00001)	188 (2))	The lower score, the more improvement
**Herbal medicine**					
CQ8.	Herbal medicine	UCT	RR 1.19	532	The higher score,

Total clinical effective rate (5, RCTs) [76, 79-82]	(270)	(262)	(1.11, 1.28, p<0.00001)	(5)	the more effect
Pain (3, RCTs) [76-78]	Herbal medicine<br>(127)y	UCT<br>(125)	MD -0.55<br>(-0.70,-0.40, p<0.00001)	252<br>(3)	The lower score,<br>the lower pain intensity
Function (ODI) (3, RCTs)[76-78]	Herbal medicine<br>(127)	UCT<br>(125)	MD -3.86<br>(-4.71,-3.02, p<0.00001)	252<br>(3)	The lower score,<br>the more improvement
Function (JOA) (1, RCTs)[79]	Herbal medicine<br>(38)	UCT<br>(38)	MD 1.46<br>(0.95,1.97, p<0.00001)	76<br>(1)	The higher score,<br>the more improvement
CQ9.<br>Total clinical effective rate (21, RCTs) [40,41,83,85-100,102-103]	Herbal medicine + UCT<br>(1369)	UCT (1290)	RR 1.32<br>(1.26, 1.37, p<0.00001)	2,659<br>(21)	The higher score,<br>the more effect
Pain (10, RCTs)[83,84,94,97-103]	Herbal medicine + UCT<br>(485)	UCT<br>(483)	MD -1.51<br>(-1.57,-1.46, p<0.00001)	968<br>(10)	The lower score,<br>the lower pain intensity
Function (ODI) (1, RCTs)[83]	Herbal medicine + UCT<br>(40)	UCT<br>(40)	MD -5.25<br>(-8.33,-2.17, p=0.0008)	80<br>(1)	The lower score,<br>the more improvement

Function (JOA) (4, RCTs)[85,97,98,101]	Herbal medicine + UCT (219)	UCT (217)	MD 5.89 (5.31,6.46, p<0.00001)	436 (4)	The higher score, the more improvement
**Pharmacopuncture**					
CQ10. Total clinical effective rate (5, RCTs)[97, 105, 107, 109]	Pharmacopuncture + UCT (142)	UCT (145)	RR 1.19 (1.07, 1.32, P=0.001)	287 (5)	The higher score, the more effect
Pain (8, RCTs)[97, 104-109]	Pharmacopuncture + UCT (191)	UCT (191)	MD -1.65 (-1.70,-1.61, p<0.00001)	382 (8)	The lower score, the lower pain intensity
Function (ODI) (1, RCTs)[110]	Pharmacopuncture + UCT (16)	UCT (13)	MD -8.39 (-10.50,-6.28,p<0.00001)	29 (1)	The lower score, the more improvement
Function (ODIc) (3, RCTs) [104, 106, 109]	Pharmacopuncture + UCT (44)	UCT (46)	MD 6.22 (3.10,9.33, p<0.0001)	90 (3)	The higher score, the more improvement

Function (JOA) (1, RCTs) [97]	Pharmacopuncture + UCT (62)	UCT (60)	MD 9.00 (7.89,10.11, p<0.00001)	122 (1)	The higher score, the more improvement
**Chuna manual therapy**					
CQ11. Total clinical effective rate (10, RCTs) [111-120]	Chuna manual therapy (639)	UCT (693)	RR 1.17 (1.12, 1.23, p<0.00001)	1,332 (10)	The higher score, the more effect
Pain (5, RCTs) [111-115]	Chuna manual therapy (389)	UCT (374)	MD -1.09 (-1.32,-0.86, p<0.00001)	763 (5)	The lower score, the lower pain intensity
Function (ODI) (1, RCTs) [113]	Chuna manual therapy (60)	UCT (60)	MD -9.87 (-15.68,-4.06, p=0.0009)	120 (1)	The lower score, the more improvement
Function (JOA) (1, RCTs) [116]	Chuna manual therapy (36)	UCT (30)	MD 4.85 (3.87,5.83, p<0.00001)	66 (1)	The higher score, the more improvement
CQ12. Total clinical effective rate (29, RCTs)[36, 39, 89, 99, 121, 124-147]	Chuna manual therapy + UCT (1970)	UCT (1785)	RR 1.25 (1.22, 1.29, p<0.00001)	3,755 (29)	The higher score, the more effect

Pain (8, RCTs) [36, 99, 121-126]	Chuna manual therapy + UCT (382)	UCT (380)	MD -1.08 (-1.21,-0.95, p<0.00001)	762 (8)	The lower score, the lower pain intensity
Function (ODI) (2, RCTs) [36, 124]	Chuna manual therapy + UCT (103)	UCT (103)	MD -2.93 (-3.38,-2.49, p<0.00001)	206 (2)	The lower score, the more improvement
Function (JOA) (4, RCTs) [46, 124, 127, 128]	Chuna manual therapy + UCT (206)	UCT (216)	MD 4.86 (4.19,5.53, p<0.00001)	422 (4)	The higher score, the more improvement
**Thread-embedding acupuncture**					
CQ13. Total clinical effective rate (12, RCTs) [148-159]	Thread-embedding acupuncture (695)	UCT (659)	RR 1.14 (1.10, 1.19, p<0.00001)	1,354 (12)	The higher score, the more effect
Pain (4, RCTs) [148-151]	Thread-embedding acupuncture (110)	UCT (110)	MD -0.40 (-0.54,-0.26, p<0.00001)	220 (4)	The lower score, the lower pain intensity
Function (ODI) (1, RCTs)[151]	Thread-embedding acupuncture (30)	UCT (30)	MD -1.30 (-2.42,-0.18, p=0.02)	60 (1)	The lower score, the more improvement

Function (JOA) (1, RCTs)[148]	Thread-embedding acupuncture (30)	UCT (30)	MD 2.03 (0.30,3.76, p=0.02)	60 (1)	The higher score, the more improvement
CQ14. Total clinical effective rate (6, RCTs) [160, 162-166]	Thread-embedding acupuncture + aUCT (470)	UCT (420)	RR 1.15 (1.09, 1.21, p<0.00001)	890 (6)	The higher score, the more effect
Pain (2, RCTs))[160, 161]	Thread-embedding acupuncture + UCT (65)	UCT (65)	MD -1.75 (-2.16,-1.33, p<0.00001)	130 (2)	The lower score, the lower pain intensity
Function (ODI) (2, RCTs) [160, 161]	Thread-embedding acupuncture + UCT (65)	UCT (65)	MD -12.94 (-17.25,-8.59,p<0.00001)	130 (2)	The lower score, the more improvement
Function (JOA) (1, RCTs) [160]	Thread-embedding acupuncture + UCT (30)	UCT (30)	MD 2.37 (0.78,3.96, p=0.004)	60 (1)	The higher score, the more improvement

**Cupping**					
CQ15.<br>Total clinical effective rate (5, RCTs) [167-171]	Cupping + UCT<br>(175)	UCT<br>(173)	RR 1.43<br>(1.27, 1.62, p<0.00001)	348<br>(5)	The higher score,<br>the more effect
Pain (1, RCTs)[167]	Cupping + UCT<br>(30)	UCT<br>(30)	MD -1.08<br>(-2.24,-0.08, p=0.07)	60<br>(1)	The lower score,<br>the lower pain intensity

UCT, Usual conventional treatment; MD, mean difference; RR, risk ratio;
