# Supplementary material for: Korean Medicine Clinical Practice Guidelines for Lumbar Herniated Intervertebral Disc in Adults: Based on Grading of Recommendations Assessment, Development and Evaluation (GRADE)
Source: Healthcare (Basel). 2022 Jan 27;10(2):246. doi: 10.3390/healthcare10020246 (PMC8871943; doi:10.3390/healthcare10020246)

Acupuncture vs. active control treatment

Total clinical effective rate

Study or Subgroup	Experimental Events	Experimental Total	Control Events	Control Total	Weight	Risk Ratio M-H, Fixed, 95% CI
**1.3.1 vs Western medicine**						
Chen MR (2009)	27	30	22	30	2.5%	1.23 [0.96, 1.57]
Du Z (2009)	29	32	25	30	2.9%	1.09 [0.89, 1.32]
Feng H (2012)	36	40	24	40	2.7%	1.50 [1.14, 1.97]
Geng X (2009)	49	56	36	52	4.2%	1.26 [1.03, 1.55]
Hou SB (2009)	26	30	17	30	1.9%	1.53 [1.09, 2.16]
Li YQ (2006)	38	40	28	37	3.2%	1.26 [1.03, 1.53]
Shan YL (2011)	38	45	26	40	3.1%	1.30 [1.00, 1.68]
Wang XG (2008)	50	52	37	52	4.1%	1.35 [1.13, 1.62]
Wu YC (2004)	160	200	76	100	11.3%	1.05 [0.92, 1.20]
Zhang BM (2008)	85	98	72	96	8.1%	1.16 [1.01, 1.33]
**Subtotal (95% CI)**		**623**		**507**	**43.9%**	**1.21 [1.14, 1.29]**
Total events	538		363			
Heterogeneity: Chi² = 12.19, df = 9 (P = 0.20); I² = 26%						
Test for overall effect: Z = 6.07 (P < 0.00001)						
**1.3.3 vs Injection**						
Chen MR (2009)	27	30	19	30	2.1%	1.42 [1.06, 1.91]
Ding WC (2014)	73	75	58	75	6.5%	1.26 [1.11, 1.43]
Xia SY (2018)	53	55	46	55	5.1%	1.15 [1.01, 1.31]
**Subtotal (95% CI)**		**160**		**160**	**13.7%**	**1.24 [1.14, 1.36]**
Total events	153		123			
Heterogeneity: Chi² = 2.19, df = 2 (P = 0.34); I² = 9%						
Test for overall effect: Z = 4.72 (P < 0.00001)						
**1.3.5 vs Physiotherapy**						
Fan Y (2009)	42	45	32	37	3.9%	1.08 [0.93, 1.25]
Gao H (2007)	55	60	47	60	5.2%	1.17 [1.00, 1.36]
Hu JZ (2014)	38	41	30	41	3.3%	1.27 [1.03, 1.55]
Huang CH (2007)	23	30	16	30	1.8%	1.44 [0.97, 2.12]
Li LX (2006)	79	86	58	68	7.2%	1.08 [0.96, 1.21]
Liu DM (2018)	22	22	16	21	1.9%	1.30 [1.02, 1.67]
Ma LX (2010)	96	106	73	103	8.3%	1.28 [1.11, 1.47]
Tang SD (2009)	42	46	39	47	4.3%	1.10 [0.94, 1.29]
Yin Q (2018)	42	45	35	45	3.9%	1.20 [1.01, 1.43]
Zhang JH (2004)	25	30	23	30	2.6%	1.09 [0.84, 1.40]
**Subtotal (95% CI)**		**511**		**482**	**42.4%**	**1.18 [1.12, 1.25]**
Total events	464		369			
Heterogeneity: Chi² = 8.36, df = 9 (P = 0.50); I² = 0%						
Test for overall effect: Z = 5.87 (P < 0.00001)						
**Total (95% CI)**		**1294**		**1149**	**100.0%**	**1.20 [1.16, 1.25]**
Total events	1155		855			
Heterogeneity: Chi² = 23.92, df = 22 (P = 0.35); I² = 8%						
Test for overall effect: Z = 9.52 (P < 0.00001)						
Test for subgroup differences: Chi² = 0.95, df = 2 (P = 0.62), I² = 0%						

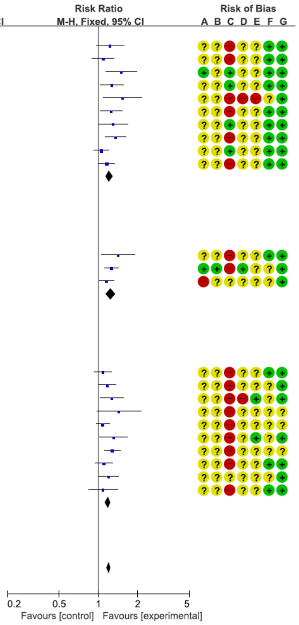

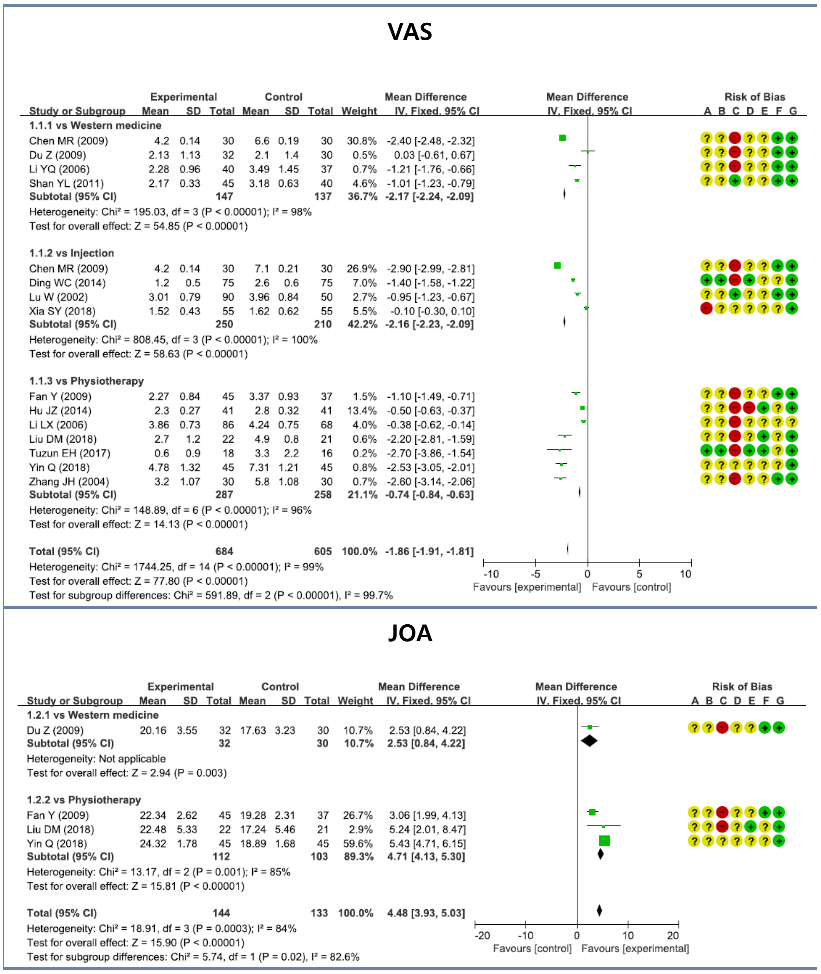
VAS
Experimental
Control
Mean Difference
Mean Difference
Risk of Bias
Study or Subgroup Mean SD Total Mean SD Total Weight IV, Fixed, 95% CI
IV, Fixed, 95% CI
A B C D E F G
1.1.1 vs Western medicine
Chen MR (2009) 4.2 0.14 30 6.6 0.19 30 30.8% -2.40 [-2.48, -2.32]
Du Z (2009) 2.13 1.13 32 2.1 1.4 30 0.5% 0.03 [-0.61, 0.67]
Li YQ (2006) 2.28 0.96 40 3.49 1.45 37 0.7% -1.21 [-1.76, -0.66]
Shan YL (2011) 2.17 0.33 45 3.18 0.63 40 4.6% -1.01 [-1.23, -0.79]
Subtotal (95% CI) 147 137 36.7% -2.17 [-2.24, -2.09]
Heterogeneity: Chi² = 195.03, df = 3 (P < 0.00001); I² = 98%
Test for overall effect: Z = 54.85 (P < 0.00001)
1.1.2 vs Injection
Chen MR (2009) 4.2 0.14 30 7.1 0.21 30 26.9% -2.90 [-2.99, -2.81]
Ding WC (2014) 1.2 0.5 75 2.6 0.6 75 7.0% -1.40 [-1.58, -1.22]
Lu W (2002) 3.01 0.79 90 3.96 0.84 50 2.7% -0.95 [-1.23, -0.67]
Xia SY (2018) 1.52 0.43 55 1.62 0.62 55 5.5% -0.10 [-0.30, 0.10]
Subtotal (95% CI) 250 210 42.2% -2.16 [-2.23, -2.09]
Heterogeneity: Chi² = 808.45, df = 3 (P < 0.00001); I² = 100%
Test for overall effect: Z = 58.63 (P < 0.00001)
1.1.3 vs Physiotherapy
Fan Y (2009) 2.27 0.84 45 3.37 0.93 37 1.5% -1.10 [-1.49, -0.71]
Hu JZ (2014) 2.3 0.27 41 2.8 0.32 41 13.4% -0.50 [-0.63, -0.37]
Li LX (2006) 3.86 0.73 86 4.24 0.75 68 4.0% -0.38 [-0.62, -0.14]
Liu DM (2018) 2.7 1.2 22 4.9 0.8 21 0.6% -2.20 [-2.81, -1.59]
Tuzun EH (2017) 0.6 0.9 18 3.3 2.2 16 0.2% -2.70 [-3.86, -1.54]
Yin Q (2018) 4.78 1.32 45 7.31 1.21 45 0.8% -2.53 [-3.05, -2.01]
Zhang JH (2004) 3.2 1.07 30 5.8 1.08 30 0.7% -2.60 [-3.14, -2.06]
Subtotal (95% CI) 287 258 21.1% -0.74 [-0.84, -0.63]
Heterogeneity: Chi² = 148.89, df = 6 (P < 0.00001); I² = 96%
Test for overall effect: Z = 14.13 (P < 0.00001)
Total (95% CI) 684 605 100.0% -1.86 [-1.91, -1.81]
Heterogeneity: Chi² = 1744.25, df = 14 (P < 0.00001); I² = 99%
Test for overall effect: Z = 77.80 (P < 0.00001)
Test for subgroup differences: Chi² = 591.89, df = 2 (P < 0.00001), I² = 99.7%
-10 -5 0 5 10
Favours [experimental] Favours [control]
JOA
Experimental
Control
Mean Difference
Mean Difference
Risk of Bias
Study or Subgroup Mean SD Total Mean SD Total Weight IV, Fixed, 95% CI
IV, Fixed, 95% CI
A B C D E F G
1.2.1 vs Western medicine
Du Z (2009) 20.16 3.55 32 17.63 3.23 30 10.7% 2.53 [0.84, 4.22]
Subtotal (95% CI) 32 30 10.7% 2.53 [0.84, 4.22]
Heterogeneity: Not applicable
Test for overall effect: Z = 2.94 (P = 0.003)
1.2.2 vs Physiotherapy
Fan Y (2009) 22.34 2.62 45 19.28 2.31 37 26.7% 3.06 [1.99, 4.13]
Liu DM (2018) 22.48 5.33 22 17.24 5.46 21 2.9% 5.24 [2.01, 8.47]
Yin Q (2018) 24.32 1.78 45 18.89 1.68 45 59.6% 5.43 [4.71, 6.15]
Subtotal (95% CI) 112 103 89.3% 4.71 [4.13, 5.30]
Heterogeneity: Chi² = 13.17, df = 2 (P = 0.001); I² = 85%
Test for overall effect: Z = 15.81 (P < 0.00001)
Total (95% CI) 144 133 100.0% 4.48 [3.93, 5.03]
Heterogeneity: Chi² = 18.91, df = 3 (P = 0.0003); I² = 84%
Test for overall effect: Z = 15.90 (P < 0.00001)
Test for subgroup differences: Chi² = 5.74, df = 1 (P = 0.02), I² = 82.6%
-20 -10 0 10 20
Favours [control] Favours [experimental]

Acupuncture + usual conventional therapy vs. usual conventional therapy

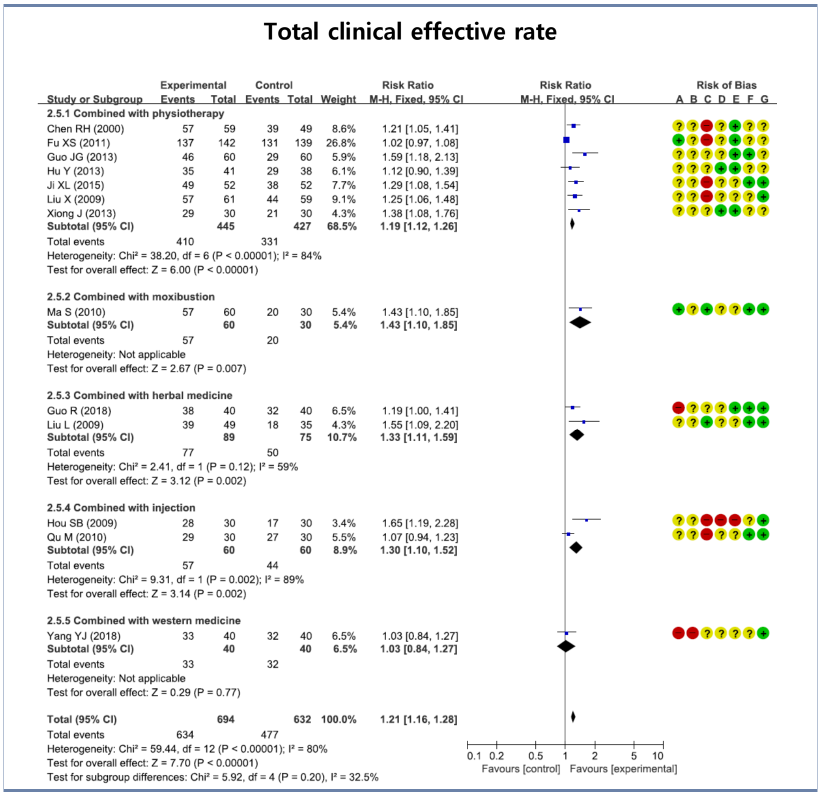

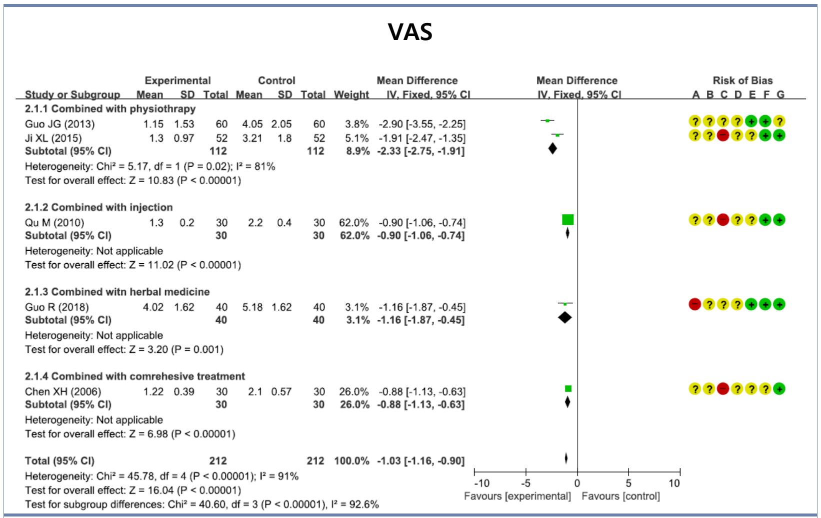
VAS
Experimental
Control
Mean Difference
Mean Difference
Risk of Bias
Study or Subgroup Mean SD Total Mean SD Total Weight IV, Fixed, 95% CI
IV, Fixed, 95% CI
A B C D E F G
2.1.1 Combined with physiothrapy
Guo JG (2013) 1.15 1.53 60 4.05 2.05 60 3.8% -2.90 [-3.55, -2.25]
Ji XL (2015) 1.3 0.97 52 3.21 1.8 52 5.1% -1.91 [-2.47, -1.35]
Subtotal (95% CI) 112 112 8.9% -2.33 [-2.75, -1.91]
Heterogeneity: Chi² = 5.17, df = 1 (P = 0.02); I² = 81%
Test for overall effect: Z = 10.83 (P < 0.00001)
2.1.2 Combined with injection
Qu M (2010) 1.3 0.2 30 2.2 0.4 30 62.0% -0.90 [-1.06, -0.74]
Subtotal (95% CI) 30 30 62.0% -0.90 [-1.06, -0.74]
Heterogeneity: Not applicable
Test for overall effect: Z = 11.02 (P < 0.00001)
2.1.3 Combined witn herbal medicine
Guo R (2018) 4.02 1.62 40 5.18 1.62 40 3.1% -1.16 [-1.87, -0.45]
Subtotal (95% CI) 40 40 3.1% -1.16 [-1.87, -0.45]
Heterogeneity: Not applicable
Test for overall effect: Z = 3.20 (P = 0.001)
2.1.4 Combined with comrehesive treatment
Chen XH (2006) 1.22 0.39 30 2.1 0.57 30 26.0% -0.88 [-1.13, -0.63]
Subtotal (95% CI) 30 30 26.0% -0.88 [-1.13, -0.63]
Heterogeneity: Not applicable
Test for overall effect: Z = 6.98 (P < 0.00001)
Total (95% CI) 212 212 100.0% -1.03 [-1.16, -0.90]
Heterogeneity: Chi² = 45.78, df = 4 (P < 0.00001); I² = 91%
Test for overall effect: Z = 16.04 (P < 0.00001)
Test for subgroup differences: Chi² = 40.60, df = 3 (P < 0.00001), I² = 92.6%
-10 -5 0 5 10
Favours [experimental] Favours [control]

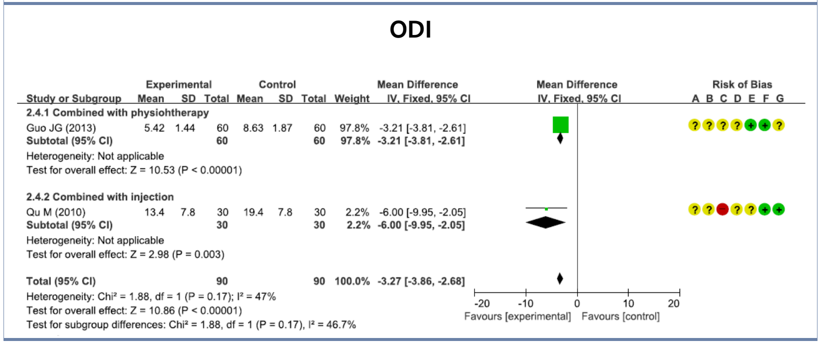
ODI
Experimental
Control
Mean Difference
Mean Difference
Risk of Bias
Study or Subgroup Mean SD Total Mean SD Total Weight IV, Fixed, 95% CI
IV, Fixed, 95% CI
A B C D E F G
2.4.1 Combined with physiohtherapy
Guo JG (2013) 5.42 1.44 60 8.63 1.87 60 97.8% -3.21 [-3.81, -2.61]
Subtotal (95% CI) 60 60 97.8% -3.21 [-3.81, -2.61]
Heterogeneity: Not applicable
Test for overall effect: Z = 10.53 (P < 0.00001)
2.4.2 Combined with injection
Qu M (2010) 13.4 7.8 30 19.4 7.8 30 2.2% -6.00 [-9.95, -2.05]
Subtotal (95% CI) 30 30 2.2% -6.00 [-9.95, -2.05]
Heterogeneity: Not applicable
Test for overall effect: Z = 2.98 (P = 0.003)
Total (95% CI) 90 90 100.0% -3.27 [-3.86, -2.68]
Heterogeneity: Chi² = 1.88, df = 1 (P = 0.17); I² = 47%
Test for overall effect: Z = 10.86 (P < 0.00001)
Test for subgroup differences: Chi² = 1.88, df = 1 (P = 0.17), I² = 46.7%
-20 -10 0 10 20
Favours [experimental] Favours [control]

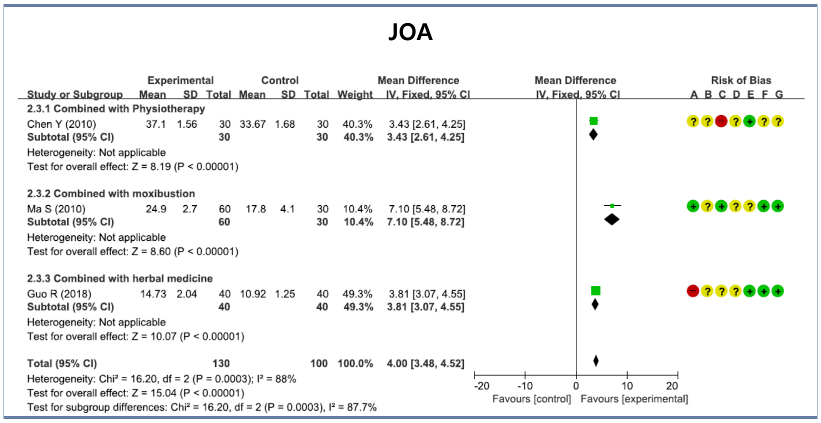
JOA
Experimental
Control
Mean Difference
Mean Difference
Risk of Bias
Study or Subgroup
Mean
SD
Total
Mean
SD
Total
Weight
IV, Fixed, 95% CI
IV, Fixed, 95% CI
A B C D E F G
2.3.1 Combined with Physiotherapy
Chen Y (2010) 37.1 1.56 30 33.67 1.68 30 40.3% 3.43 [2.61, 4.25]
Subtotal (95% CI) 30 30 40.3% 3.43 [2.61, 4.25]
Heterogeneity: Not applicable
Test for overall effect: Z = 8.19 (P < 0.00001)
2.3.2 Combined with moxibustion
Ma S (2010) 24.9 2.7 60 17.8 4.1 30 10.4% 7.10 [5.48, 8.72]
Subtotal (95% CI) 60 30 10.4% 7.10 [5.48, 8.72]
Heterogeneity: Not applicable
Test for overall effect: Z = 8.60 (P < 0.00001)
2.3.3 Combined with herbal medicine
Guo R (2018) 14.73 2.04 40 10.92 1.25 40 49.3% 3.81 [3.07, 4.55]
Subtotal (95% CI) 40 40 49.3% 3.81 [3.07, 4.55]
Heterogeneity: Not applicable
Test for overall effect: Z = 10.07 (P < 0.00001)
Total (95% CI) 130 100 100.0% 4.00 [3.48, 4.52]
Heterogeneity: Chi² = 16.20, df = 2 (P = 0.0003); I² = 88%
Test for overall effect: Z = 15.04 (P < 0.00001)
Test for subgroup differences: Chi² = 16.20, df = 2 (P = 0.0003), I² = 87.7%
-20
-10
0
10
20
Favours [control]
Favours [experimental]

Electro-acupuncture, fire needling, or warm needling vs. acupuncture

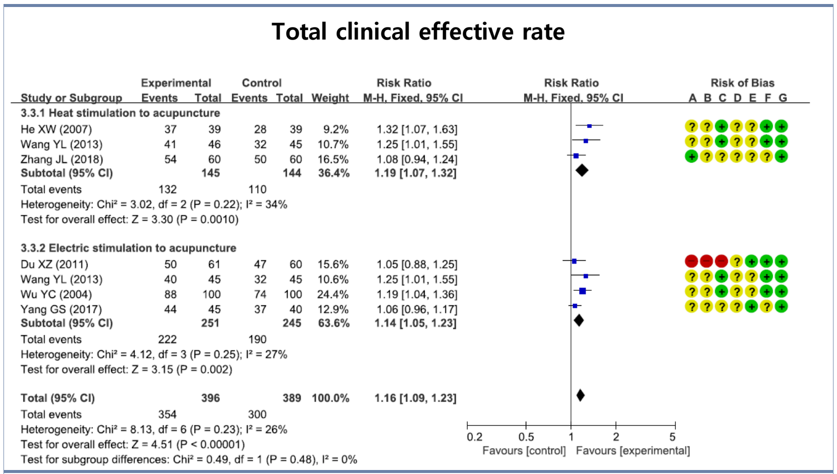

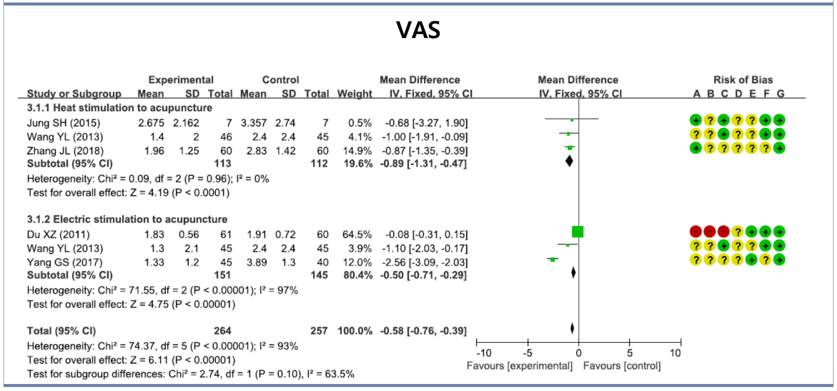

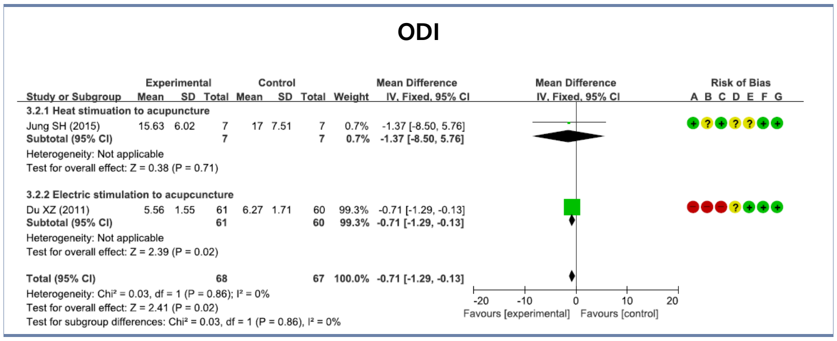
ODI
Study or Subgroup
Experimental
Mean SD Total
Control
Mean SD Total
Weight
Mean Difference
IV, Fixed, 95% CI
Mean Difference
IV, Fixed, 95% CI
Risk of Bias
A B C D E F G
3.2.1 Heat stimulation to acupuncture
Jung SH (2015) 15.63 6.02 7 17 7.51 7 0.7% -1.37 [-8.50, 5.76]
Subtotal (95% CI) 7 7 0.7% -1.37 [-8.50, 5.76]
Heterogeneity: Not applicable
Test for overall effect: Z = 0.38 (P = 0.71)
3.2.2 Electric stimulation to acupuncture
Du XZ (2011) 5.56 1.55 61 6.27 1.71 60 99.3% -0.71 [-1.29, -0.13]
Subtotal (95% CI) 61 60 99.3% -0.71 [-1.29, -0.13]
Heterogeneity: Not applicable
Test for overall effect: Z = 2.39 (P = 0.02)
Total (95% CI) 68 67 100.0% -0.71 [-1.29, -0.13]
Heterogeneity: Chi² = 0.03, df = 1 (P = 0.86); I² = 0%
Test for overall effect: Z = 2.41 (P = 0.02)
Test for subgroup differences: Chi² = 0.03, df = 1 (P = 0.86), I² = 0%
-20 -10 0 10 20
Favours [experimental] Favours [control]

Deep insertion acupuncture vs. superficial insertion acupuncture

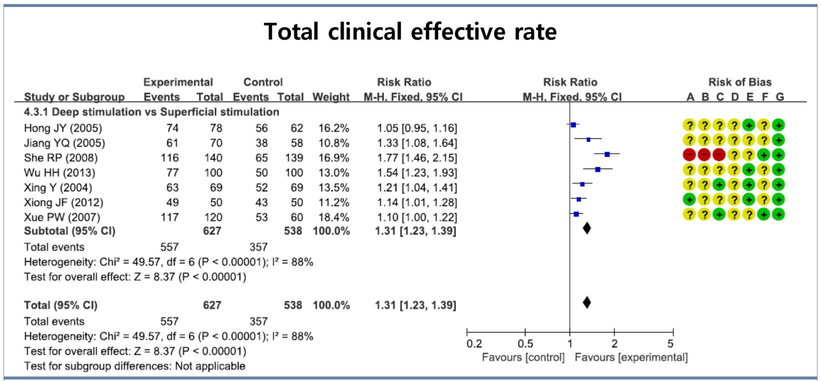

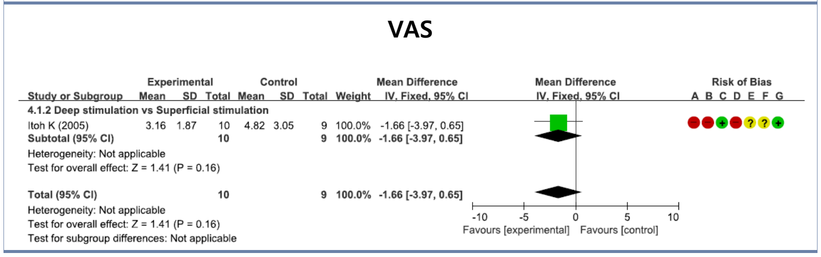

Moxibustion vs. active control group

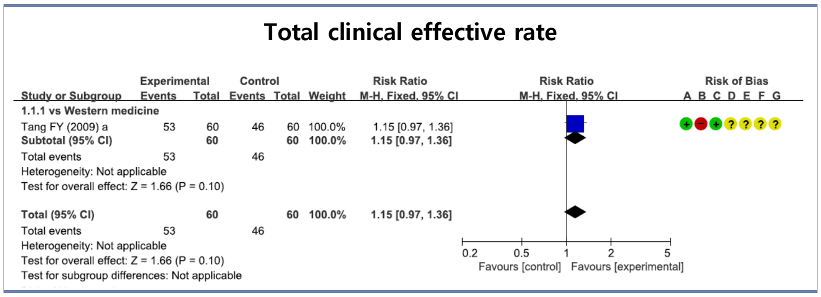

Moxibustion + usual conventional therapy vs. usual conventional therapy

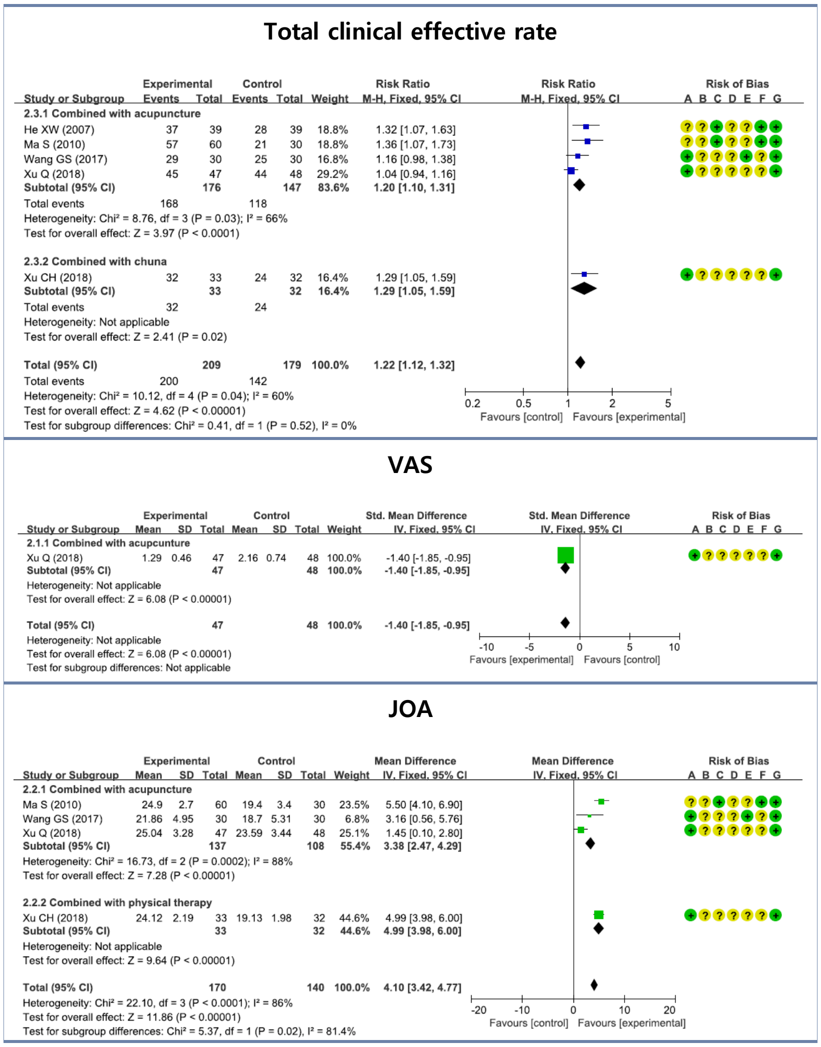

Moxibustion causing deqi sensation vs. moxibustion not causing deqi sensation

Total clinical effective rate

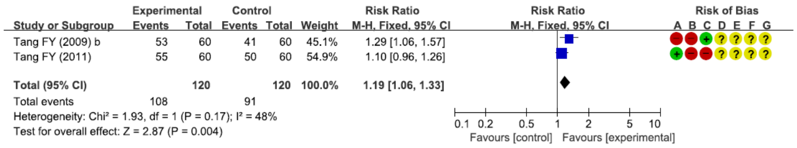

Modified JOA

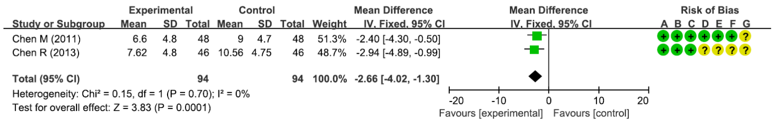

Herbal medicine vs. active control treatment

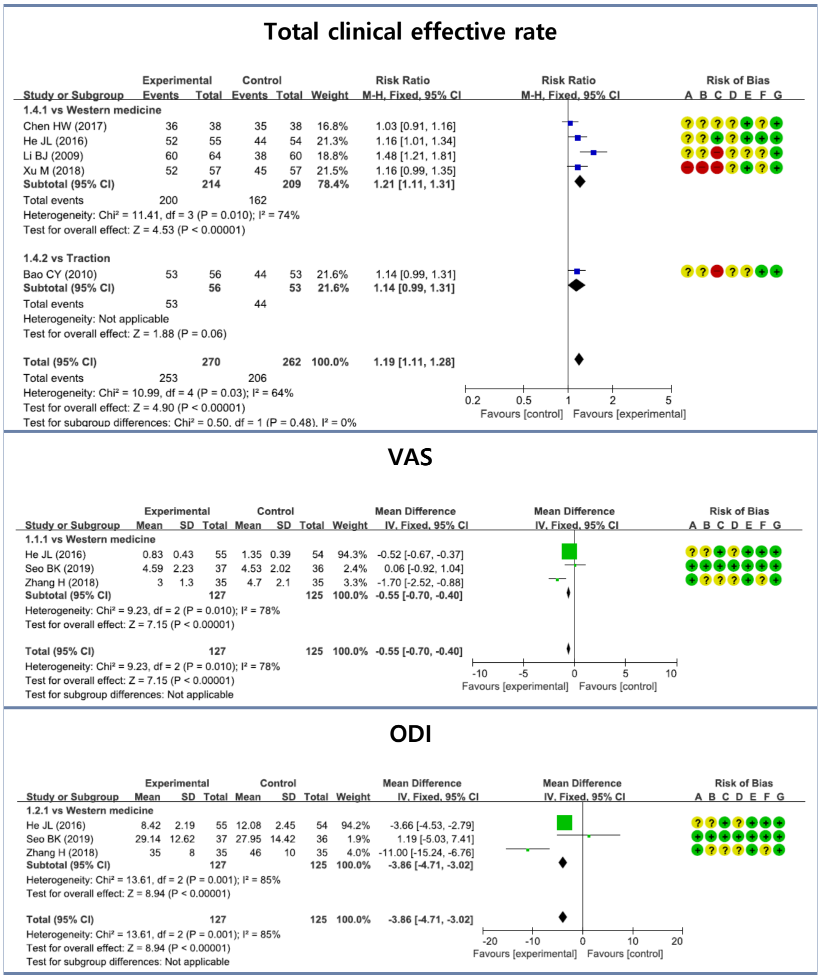

JOA

Study or Subgroup	Experimental Mean	Experimental SD	Experimental Total	Control Mean	Control SD	Control Total	Weight	Mean Difference IV, Fixed, 95% CI
**1.3.1 vs Western medicine**								
Chen HW (2017)	17.56	0.96	38	16.1	1.28	38	100.0%	1.46 [0.95, 1.97]
**Subtotal (95% CI)**			**38**			**38**	**100.0%**	**1.46 [0.95, 1.97]**
Heterogeneity: Not applicable								
Test for overall effect: Z = 5.63 (P < 0.00001)								
**Total (95% CI)**			**38**			**38**	**100.0%**	**1.46 [0.95, 1.97]**
Heterogeneity: Not applicable								
Test for overall effect: Z = 5.63 (P < 0.00001)								
Test for subgroup differences: Not applicable								

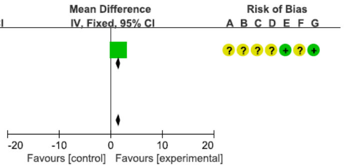

Herbal medicine + usual conventional therapy vs. usual conventional therapy

Total clinical effective rate

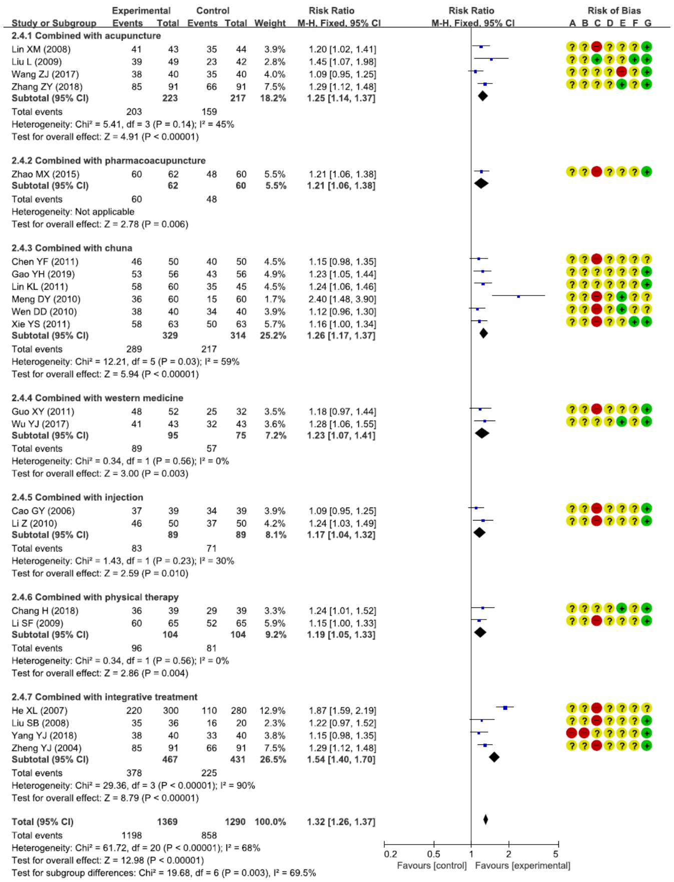

VAS

Study or Subgroup	Experimental Mean	Experimental SD	Experimental Total	Control Mean	Control SD	Control Total	Weight	Mean Difference IV, Fixed, 95% CI
**2.1.1 Combined with acupuncture**								
Wang ZJ (2017)	1.98	0.83	40	3.48	1.14	40	1.5%	-1.50 [-1.94, -1.06]
Zhang ZY (2018)	3.94	1.51	48	5.11	1.78	48	0.7%	-1.17 [-1.83, -0.51]
**Subtotal (95% CI)**			**88**			**88**	**2.2%**	**-1.40 [-1.76, -1.04]**
Heterogeneity: Chi² = 0.67, df = 1 (P = 0.41); I² = 0%								
Test for overall effect: Z = 7.53 (P < 0.00001)								
**2.1.2 Combined with pharmacoacupuncture**								
Zhao MX (2015)	1.56	0.14	62	3.12	0.21	60	71.9%	-1.56 [-1.62, -1.50]
**Subtotal (95% CI)**			**62**			**60**	**71.9%**	**-1.56 [-1.62, -1.50]**
Heterogeneity: Not applicable								
Test for overall effect: Z = 48.12 (P < 0.00001)								
**2.1.3 Combined with chuna**								
Gao YH (2019)	1.93	0.56	56	3.05	0.82	56	4.3%	-1.12 [-1.38, -0.86]
Wen DD (2010)	0.78	0.24	40	1.64	0.65	40	6.3%	-0.86 [-1.07, -0.65]
Xie YS (2011)	1.2	0.3	63	3.21	0.9	63	5.3%	-2.01 [-2.24, -1.78]
**Subtotal (95% CI)**			**159**			**159**	**15.9%**	**-1.31 [-1.45, -1.18]**
Heterogeneity: Chi² = 53.22, df = 2 (P < 0.00001); I² = 96%								
Test for overall effect: Z = 19.04 (P < 0.00001)								
**2.1.4 Combined with western medicine**								
Miao LH (2018)	2.6	0.6	58	3.6	1.2	58	2.4%	-1.00 [-1.35, -0.65]
**Subtotal (95% CI)**			**58**			**58**	**2.4%**	**-1.00 [-1.35, -0.65]**
Heterogeneity: Not applicable								
Test for overall effect: Z = 5.68 (P < 0.00001)								
**2.1.5 Combined with injection**								
Cao GY (2006)	1.8	0.7	39	2.2	1	39	2.0%	-0.40 [-0.78, -0.02]
**Subtotal (95% CI)**			**39**			**39**	**2.0%**	**-0.40 [-0.78, -0.02]**
Heterogeneity: Not applicable								
Test for overall effect: Z = 2.05 (P = 0.04)								
**2.1.6 Combined with physical therapy**								
Chang H (2018)	2.1	0.4	39	4.9	0.8	39	3.7%	-2.80 [-3.08, -2.52]
**Subtotal (95% CI)**			**39**			**39**	**3.7%**	**-2.80 [-3.08, -2.52]**
Heterogeneity: Not applicable								
Test for overall effect: Z = 19.55 (P < 0.00001)								
**2.1.7 Combined with integrative treatment**								
Wang J (2008)	1.31	0.71	40	2.12	1.03	40	1.9%	-0.81 [-1.20, -0.42]
**Subtotal (95% CI)**			**40**			**40**	**1.9%**	**-0.81 [-1.20, -0.42]**
Heterogeneity: Not applicable								
Test for overall effect: Z = 4.10 (P < 0.0001)								
**Total (95% CI)**			**485**			**483**	**100.0%**	**-1.51 [-1.57, -1.46]**
Heterogeneity: Chi² = 199.01, df = 9 (P < 0.00001); I² = 95%								
Test for overall effect: Z = 55.00 (P < 0.00001)								
Test for subgroup differences: Chi² = 145.13, df = 6 (P < 0.00001), I² = 95.9%								

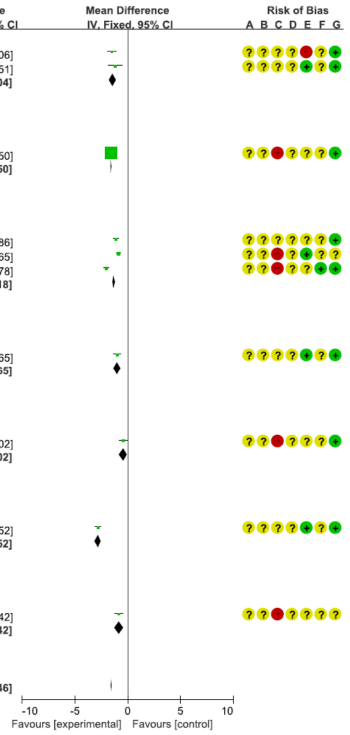

ODI

Study or Subgroup	Experimental Mean	Experimental SD	Experimental Total	Control Mean	Control SD	Control Total	Weight	Mean Difference IV, Fixed, 95% CI
**2.2.1 Combined with acupuncture**								
Wang ZJ (2017)	7.35	6.53	40	12.6	7.48	40	100.0%	-5.25 [-8.33, -2.17]
**Subtotal (95% CI)**			**40**			**40**	**100.0%**	**-5.25 [-8.33, -2.17]**
Heterogeneity: Not applicable								
Test for overall effect: Z = 3.34 (P = 0.0008)								
**Total (95% CI)**			**40**			**40**	**100.0%**	**-5.25 [-8.33, -2.17]**
Heterogeneity: Not applicable								
Test for overall effect: Z = 3.34 (P = 0.0008)								
Test for subgroup differences: Not applicable								

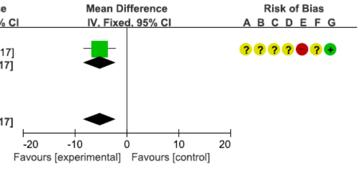

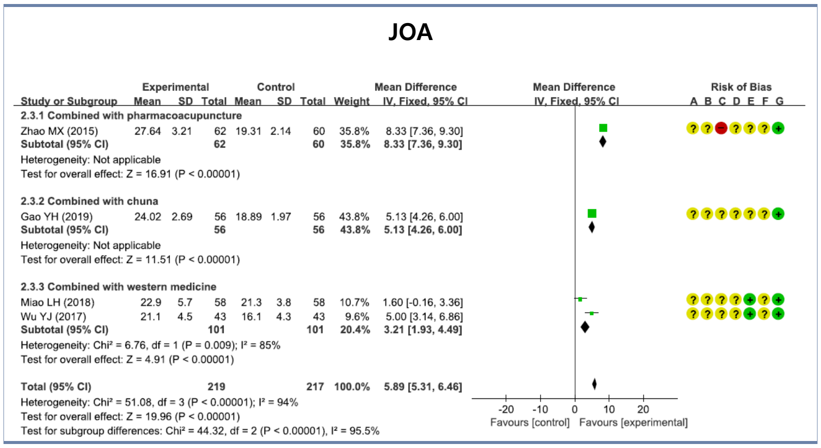

JOA
Experimental
Control
Mean Difference
Study or Subgroup
Mean
SD
Total
Mean
SD
Total
Weight
IV, Fixed, 95% CI
2.3.1 Combined with pharmacoacupuncture
Zhao MX (2015) 27.64 3.21 62 19.31 2.14 60 35.8% 8.33 [7.36, 9.30]
Subtotal (95% CI) 62 60 35.8% 8.33 [7.36, 9.30]
Heterogeneity: Not applicable
Test for overall effect: Z = 16.91 (P < 0.00001)
2.3.2 Combined with chuna
Gao YH (2019) 24.02 2.69 56 18.89 1.97 56 43.8% 5.13 [4.26, 6.00]
Subtotal (95% CI) 56 56 43.8% 5.13 [4.26, 6.00]
Heterogeneity: Not applicable
Test for overall effect: Z = 11.51 (P < 0.00001)
2.3.3 Combined with western medicine
Miao LH (2018) 22.9 5.7 58 21.3 3.8 58 10.7% 1.60 [-0.16, 3.36]
Wu YJ (2017) 21.1 4.5 43 16.1 4.3 43 9.6% 5.00 [3.14, 6.86]
Subtotal (95% CI) 101 101 20.4% 3.21 [1.93, 4.49]
Heterogeneity: Chi² = 6.76, df = 1 (P = 0.009); I² = 85%
Test for overall effect: Z = 4.91 (P < 0.00001)
Total (95% CI) 219 217 100.0% 5.89 [5.31, 6.46]
Heterogeneity: Chi² = 51.08, df = 3 (P < 0.00001); I² = 94%
Test for overall effect: Z = 19.96 (P < 0.00001)
Test for subgroup differences: Chi² = 44.32, df = 2 (P < 0.00001), I² = 95.5%
Mean Difference
IV, Fixed, 95% CI
-20
-10
0
10
20
Favours [control]
Favours [experimental]
Risk of Bias
A B C D E F G

Pharmacopuncture + usual conventional therapy vs. usual conventional therapy

Total clinical effective rate

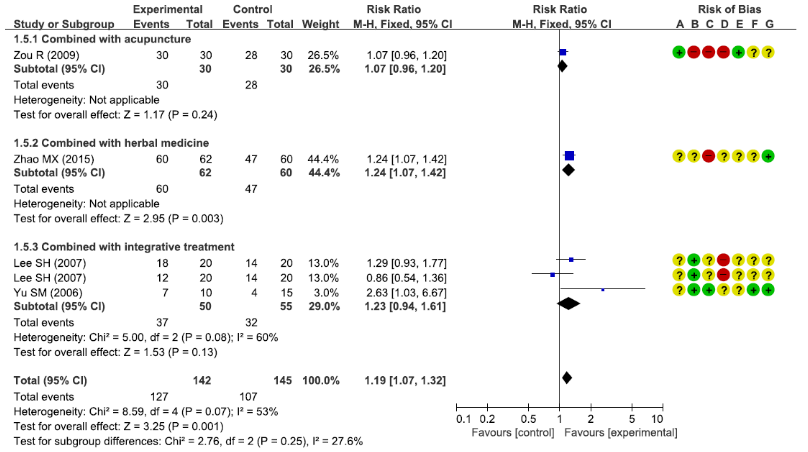

VAS

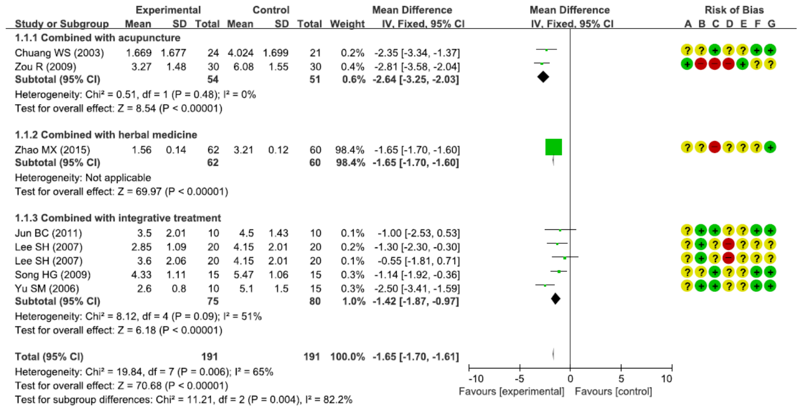

ODI

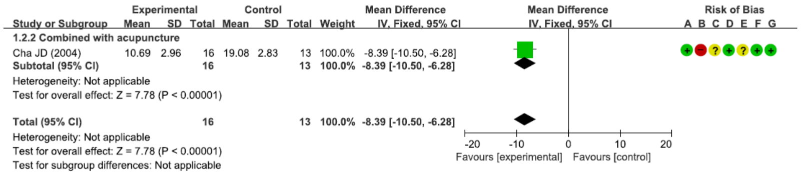

ODI change

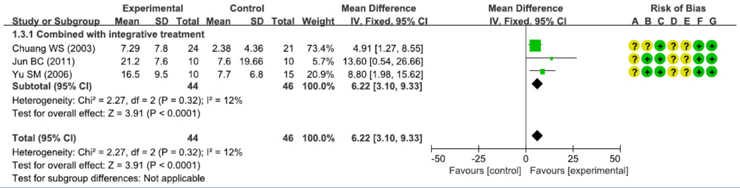

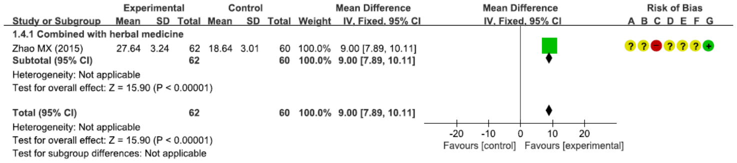

JOA

Tuina manual therapy vs active control treatment

Total clinical effective rate

Study or Subgroup	Experimental Events	Experimental Total	Control Events	Control Total	Weight	Risk Ratio M-H, Fixed, 95% CI
**1.4.1 vs Western medicine**						
Zhang L (2012)	28	28	12	20	2.8%	1.65 [1.16, 2.36]
**Subtotal (95% CI)**		**28**		**20**	**2.8%**	**1.65 [1.16, 2.36]**
Total events	28		12			
Heterogeneity: Not applicable						
Test for overall effect: Z = 2.76 (P = 0.006)						
**1.4.2 vs Injection**						
Weng WS (2008)	34	36	23	30	4.9%	1.23 [1.00, 1.52]
Zhang QM (2008)	117	126	200	219	28.7%	1.02 [0.95, 1.08]
**Subtotal (95% CI)**		**162**		**249**	**33.6%**	**1.05 [0.98, 1.12]**
Total events	151		223			
Heterogeneity: $Chi^2 = 3.10$, df = 1 (P = 0.08); $I^2 = 68\%$						
Test for overall effect: Z = 1.47 (P = 0.14)						
**1.4.3 vs Traction**						
Chen JH (2006)	108	122	83	111	17.1%	1.18 [1.04, 1.34]
Dong T (2010)	91	99	80	96	16.0%	1.10 [0.99, 1.23]
Gu F (2013)	51	60	36	60	7.1%	1.42 [1.12, 1.79]
Hu GX (2009)	57	60	45	60	8.8%	1.27 [1.08, 1.48]
Li J (2010)	28	30	15	20	3.5%	1.24 [0.95, 1.63]
Lin X (2008)	26	30	17	30	3.3%	1.53 [1.09, 2.16]
Tang XZ (2012)	46	48	39	47	7.7%	1.15 [1.00, 1.33]
**Subtotal (95% CI)**		**449**		**424**	**63.5%**	**1.22 [1.14, 1.30]**
Total events	407		315			
Heterogeneity: $Chi^2 = 7.66$, df = 6 (P = 0.26); $I^2 = 22\%$						
Test for overall effect: Z = 6.17 (P < 0.00001)						
**Total (95% CI)**		**639**		**693**	**100.0%**	**1.17 [1.12, 1.23]**
Total events	586		550			
Heterogeneity: $Chi^2 = 30.75$, df = 9 (P = 0.0003); $I^2 = 71\%$						
Test for overall effect: Z = 6.70 (P < 0.00001)						
Test for subgroup differences: $Chi^2 = 15.30$, df = 2 (P = 0.0005), $I^2 = 86.9\%$						

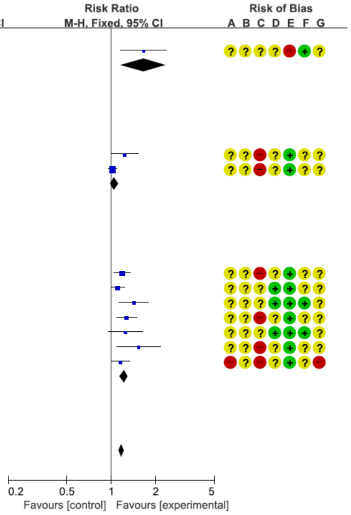

VAS

Study or Subgroup	Experimental Mean	Experimental SD	Experimental Total	Control Mean	Control SD	Control Total	Weight	Mean Difference IV, Fixed, 95% CI
**1.1.1 vs Traction**								
Chen JH (2006)	2.41	1.9	122	3.1	2.3	111	17.7%	-0.69 [-1.23, -0.15]
Dong T (2010)	3.37	1.93	99	4.35	2.01	96	17.2%	-0.98 [-1.53, -0.43]
Gu F (2013)	3.688	1.601	60	4.795	1.791	60	14.2%	-1.11 [-1.71, -0.50]
Hu GX (2009)	3.37	1.93	60	4.35	2.01	60	10.6%	-0.98 [-1.69, -0.27]
Tang XZ (2012)	2.56	0.82	48	3.89	0.97	47	40.3%	-1.33 [-1.69, -0.97]
**Subtotal (95% CI)**			**389**			**374**	**100.0%**	**-1.09 [-1.32, -0.86]**
Heterogeneity: $Chi^2 = 4.01$, df = 4 (P = 0.40); $I^2 = 0\%$								
Test for overall effect: Z = 9.29 (P < 0.00001)								
**Total (95% CI)**			**389**			**374**	**100.0%**	**-1.09 [-1.32, -0.86]**
Heterogeneity: $Chi^2 = 4.01$, df = 4 (P = 0.40); $I^2 = 0\%$								
Test for overall effect: Z = 9.29 (P < 0.00001)								
Test for subgroup differences: Not applicable								

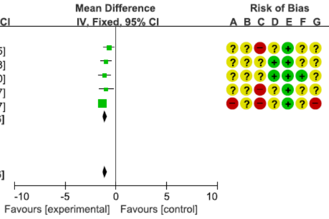

ODI

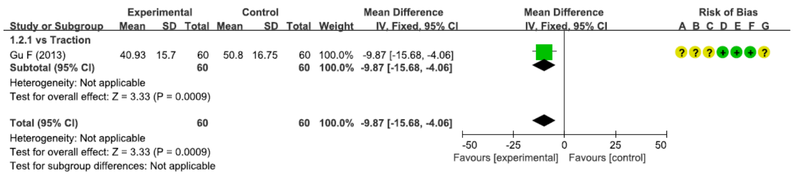

Experimental
Control
Mean Difference
Mean Difference
Risk of Bias
Study or Subgroup Mean SD Total Mean SD Total Weight IV, Fixed, 95% CI
IV, Fixed, 95% CI
A B C D E F G
1.2.1 vs Traction
Gu F (2013) 40.93 15.7 60 50.8 16.75 60 100.0% -9.87 [-15.68, -4.06]
Subtotal (95% CI) 60 60 100.0% -9.87 [-15.68, -4.06]
Heterogeneity: Not applicable
Test for overall effect: Z = 3.33 (P = 0.0009)
Total (95% CI) 60 60 100.0% -9.87 [-15.68, -4.06]
Heterogeneity: Not applicable
Test for overall effect: Z = 3.33 (P = 0.0009)
Test for subgroup differences: Not applicable
-50 -25 0 25 50
Favours [experimental] Favours [control]

JOA

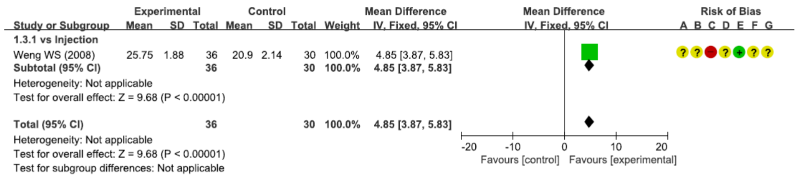

Experimental
Control
Mean Difference
Mean Difference
Risk of Bias
Study or Subgroup Mean SD Total Mean SD Total Weight IV, Fixed, 95% CI
IV, Fixed, 95% CI
A B C D E F G
1.3.1 vs Injection
Weng WS (2008) 25.75 1.88 36 20.9 2.14 30 100.0% 4.85 [3.87, 5.83]
Subtotal (95% CI) 36 30 100.0% 4.85 [3.87, 5.83]
Heterogeneity: Not applicable
Test for overall effect: Z = 9.68 (P < 0.00001)
Total (95% CI) 36 30 100.0% 4.85 [3.87, 5.83]
Heterogeneity: Not applicable
Test for overall effect: Z = 9.68 (P < 0.00001)
Test for subgroup differences: Not applicable
-20 -10 0 10 20
Favours [control] Favours [experimental]

Tuina manual therapy + usual conventional therapy vs. usual conventional therapy

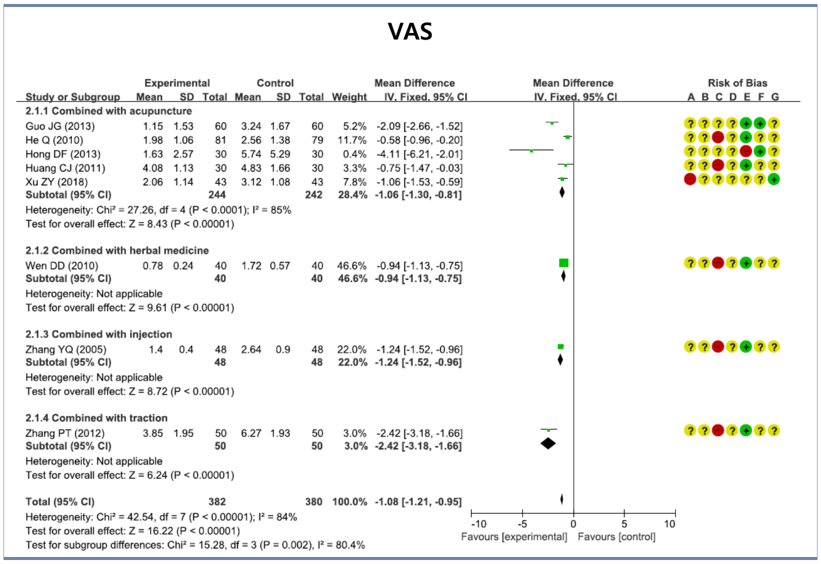

Total clinical effective rate

Study or Subgroup	Experimental Events	Experimental Total	Control Events	Control Total	Weight	Risk Ratio M-H, Fixed, 95% CI
**2.4.1 Combined with acupuncture**						
Chen RH (2000)	59	61	49	60	3.7%	1.18 [1.04, 1.35]
Chen ZL (2013)	50	60	29	60	2.2%	1.72 [1.30, 2.29]
Gao XD (2010)	43	44	30	42	2.3%	1.37 [1.12, 1.67]
Guo JG (2013)	46	60	27	60	2.0%	1.70 [1.25, 2.33]
He Q (2007)	40	45	30	53	2.1%	1.57 [1.21, 2.03]
He Q (2010)	72	81	53	79	4.0%	1.32 [1.11, 1.57]
He ZD (2009)	63	65	55	61	4.2%	1.07 [0.98, 1.18]
Xiang KW (2008)	19	20	16	20	1.2%	1.19 [0.93, 1.51]
Xiong J (2013)	29	30	21	30	1.6%	1.38 [1.08, 1.76]
Xu BJ (2013)	117	120	108	120	8.1%	1.08 [1.01, 1.16]
Xu ZY (2018)	42	43	37	43	2.8%	1.14 [1.00, 1.29]
Zeng R (2009)	26	30	22	30	1.6%	1.18 [0.91, 1.53]
**Subtotal (95% CI)**		**659**		**658**	**35.6%**	**1.27 [1.20, 1.33]**
Total events	606		477			
Heterogeneity: Chi² = 49.18, df = 11 (P < 0.00001); I² = 78%						
Test for overall effect: Z = 9.16 (P < 0.00001)						
**2.4.2 Combined with herbal medicine**						
Meng DY (2010)	36	60	19	60	1.4%	1.89 [1.24, 2.90]
Wen DD (2010)	38	40	32	40	2.4%	1.19 [1.00, 1.41]
**Subtotal (95% CI)**		**100**		**100**	**3.8%**	**1.45 [1.18, 1.78]**
Total events	74		51			
Heterogeneity: Chi² = 6.82, df = 1 (P = 0.009); I² = 85%						
Test for overall effect: Z = 3.55 (P = 0.0004)						
**2.4.3 Combined with injection**						
Ding XY (2013)	38	41	29	41	2.2%	1.31 [1.06, 1.62]
Lu CP (2013)	55	60	44	60	3.3%	1.25 [1.05, 1.48]
Yan Q (2005)	118	125	110	125	8.2%	1.07 [0.99, 1.16]
Zhang YQ (2005)	44	48	42	48	3.1%	1.05 [0.91, 1.20]
**Subtotal (95% CI)**		**274**		**274**	**16.8%**	**1.13 [1.06, 1.21]**
Total events	255		225			
Heterogeneity: Chi² = 6.22, df = 3 (P = 0.10); I² = 52%						
Test for overall effect: Z = 3.84 (P = 0.0001)						
**2.4.4 Combined with traction**						
Feng LG (2011)	76	79	66	79	4.9%	1.15 [1.03, 1.28]
Shi L (2010)	73	78	58	78	4.3%	1.26 [1.09, 1.45]
Xie TJ (2012)	42	46	33	44	2.5%	1.22 [1.00, 1.48]
Yang TH (2008)	100	100	88	100	6.6%	1.14 [1.05, 1.22]
Yuan HC (2010)	60	66	44	62	3.4%	1.28 [1.07, 1.53]
Zeng SP (2002)	74	78	60	72	4.7%	1.14 [1.01, 1.28]
Zhang JP (2009)	54	63	36	63	2.7%	1.50 [1.18, 1.90]
Zhang PT (2012)	48	50	37	50	2.8%	1.30 [1.09, 1.54]
Zhang SY (2010)	47	50	24	30	2.2%	1.18 [0.97, 1.42]
Zhang XW (2008)	233	272	53	110	5.6%	1.78 [1.46, 2.17]
Zhu J (2005)	53	55	60	65	4.1%	1.04 [0.96, 1.14]
**Subtotal (95% CI)**		**937**		**753**	**43.8%**	**1.27 [1.21, 1.34]**
Total events	860		559			
Heterogeneity: Chi² = 49.91, df = 10 (P < 0.00001); I² = 80%						
Test for overall effect: Z = 9.88 (P < 0.00001)						
**Total (95% CI)**		**1970**		**1785**	**100.0%**	**1.25 [1.22, 1.29]**
Total events	1795		1312			
Heterogeneity: Chi² = 115.97, df = 28 (P < 0.00001); I² = 76%						
Test for overall effect: Z = 14.41 (P < 0.00001)						
Test for subgroup differences: Chi² = 11.72, df = 3 (P = 0.008), I² = 74.4%						

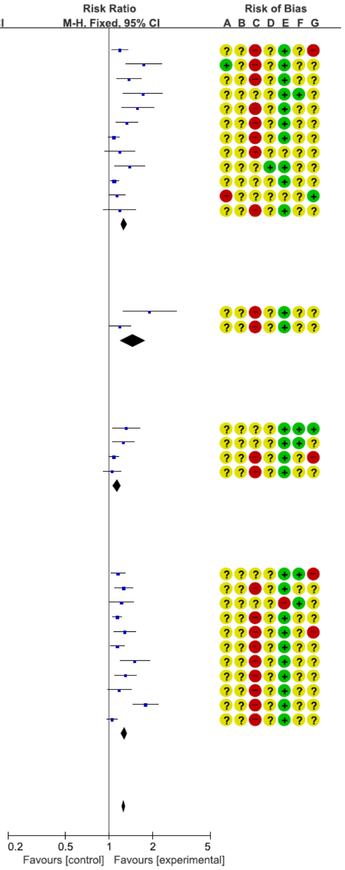

ODI

Study or Subgroup	Experimental Mean	Experimental SD	Experimental Total	Control Mean	Control SD	Control Total	Weight	Mean Difference IV, Fixed, 95% CI
**2.2.1 Combined with acupuncture**								
Guo JG (2013)	5.42	1.44	60	8.27	1.01	60	98.4%	-2.85 [-3.30, -2.40]
Xu ZY (2018)	18.81	8.39	43	27.12	8.38	43	1.6%	-8.31 [-11.85, -4.77]
**Subtotal (95% CI)**			**103**			**103**	**100.0%**	**-2.93 [-3.38, -2.49]**
Heterogeneity: Chi² = 8.97, df = 1 (P = 0.003); I² = 89%								
Test for overall effect: Z = 13.03 (P < 0.00001)								
**Total (95% CI)**			**103**			**103**	**100.0%**	**-2.93 [-3.38, -2.49]**
Heterogeneity: Chi² = 8.97, df = 1 (P = 0.003); I² = 89%								
Test for overall effect: Z = 13.03 (P < 0.00001)								
Test for subgroup differences: Not applicable								

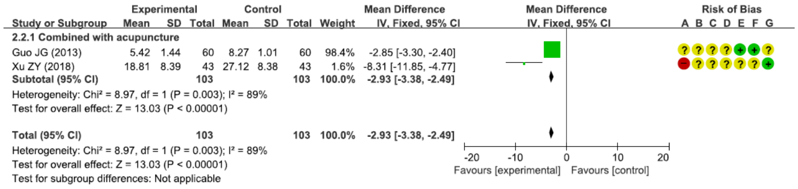

JOA

Study or Subgroup	Experimental Mean	Experimental SD	Experimental Total	Control Mean	Control SD	Control Total	Weight	Mean Difference IV, Fixed, 95% CI
**2.3.1 Combined with acupcuncture**								
Chen Y (2010)	37.1	1.56	30	33.3	2.05	30	53.0%	3.80 [2.88, 4.72]
Xu ZY (2018)	26.16	4.44	43	18.98	4.42	43	12.8%	7.18 [5.31, 9.05]
**Subtotal (95% CI)**			**73**			**73**	**65.8%**	**4.46 [3.63, 5.29]**
Heterogeneity: Chi² = 10.07, df = 1 (P = 0.002); I² = 90%								
Test for overall effect: Z = 10.57 (P < 0.00001)								
**2.3.2 Combined with traction**								
Shi L (2010)	25.31	4.11	78	20.11	4.85	78	22.6%	5.20 [3.79, 6.61]
Zhu J (2005)	25.57	4.78	55	19.12	6.25	65	11.5%	6.45 [4.47, 8.43]
**Subtotal (95% CI)**			**133**			**143**	**34.2%**	**5.62 [4.47, 6.77]**
Heterogeneity: Chi² = 1.02, df = 1 (P = 0.31); I² = 2%								
Test for overall effect: Z = 9.60 (P < 0.00001)								
**Total (95% CI)**			**206**			**216**	**100.0%**	**4.86 [4.19, 5.53]**
Heterogeneity: Chi² = 13.69, df = 3 (P = 0.003); I² = 78%								
Test for overall effect: Z = 14.18 (P < 0.00001)								
Test for subgroup differences: Chi² = 2.59, df = 1 (P = 0.11), I² = 61.4%								

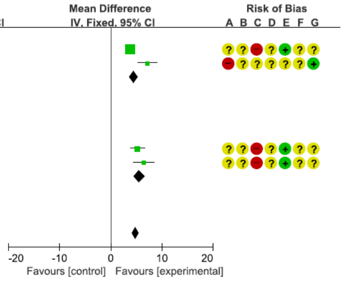

TEA vs. active control treatment

Total clinical effective rate

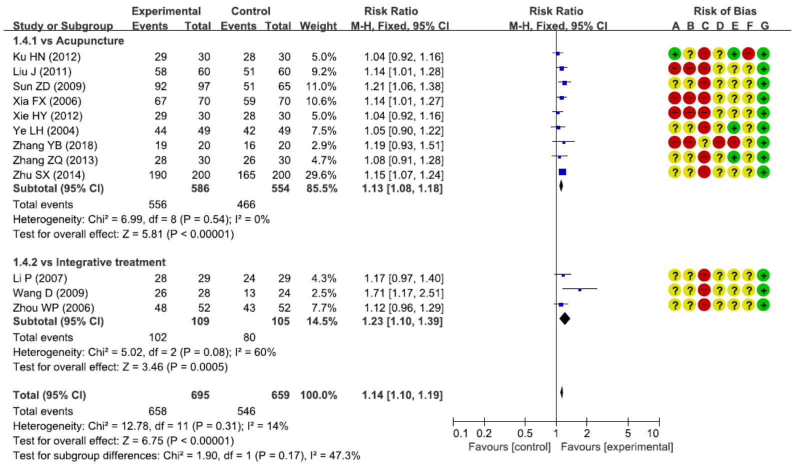

VAS

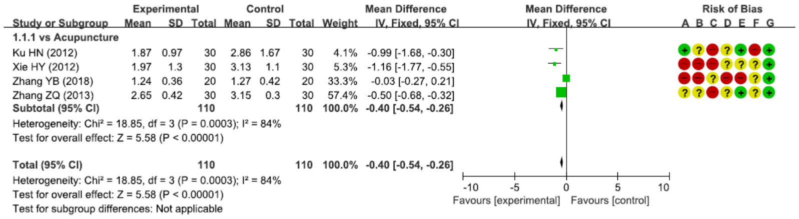

ODI

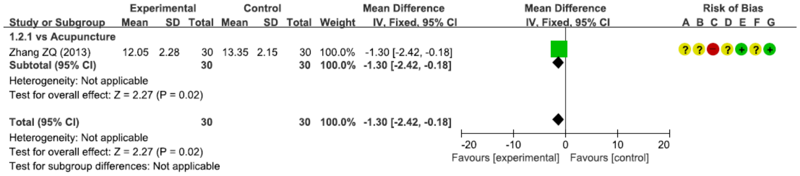

JOA

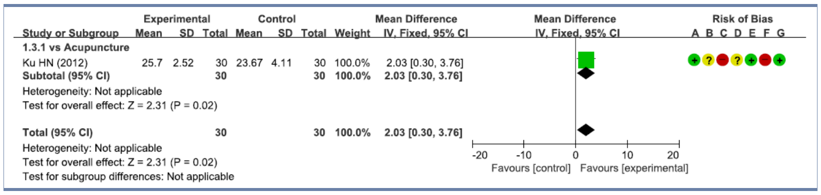
Experimental
Control
Mean Difference
Mean Difference
Risk of Bias
Study or Subgroup Mean SD Total Mean SD Total Weight IV, Fixed, 95% CI
IV, Fixed, 95% CI
A B C D E F G
1.3.1 vs Acupuncture
Ku HN (2012) 25.7 2.52 30 23.67 4.11 30 100.0% 2.03 [0.30, 3.76]
Subtotal (95% CI) 30 30 100.0% 2.03 [0.30, 3.76]
Heterogeneity: Not applicable
Test for overall effect: Z = 2.31 (P = 0.02)
Total (95% CI) 30 30 100.0% 2.03 [0.30, 3.76]
Heterogeneity: Not applicable
Test for overall effect: Z = 2.31 (P = 0.02)
Test for subgroup differences: Not applicable
-20
-10
0
10
20
Favours [control]
Favours [experimental]

TEA + usual conventional therapy vs. usual conventional therapy

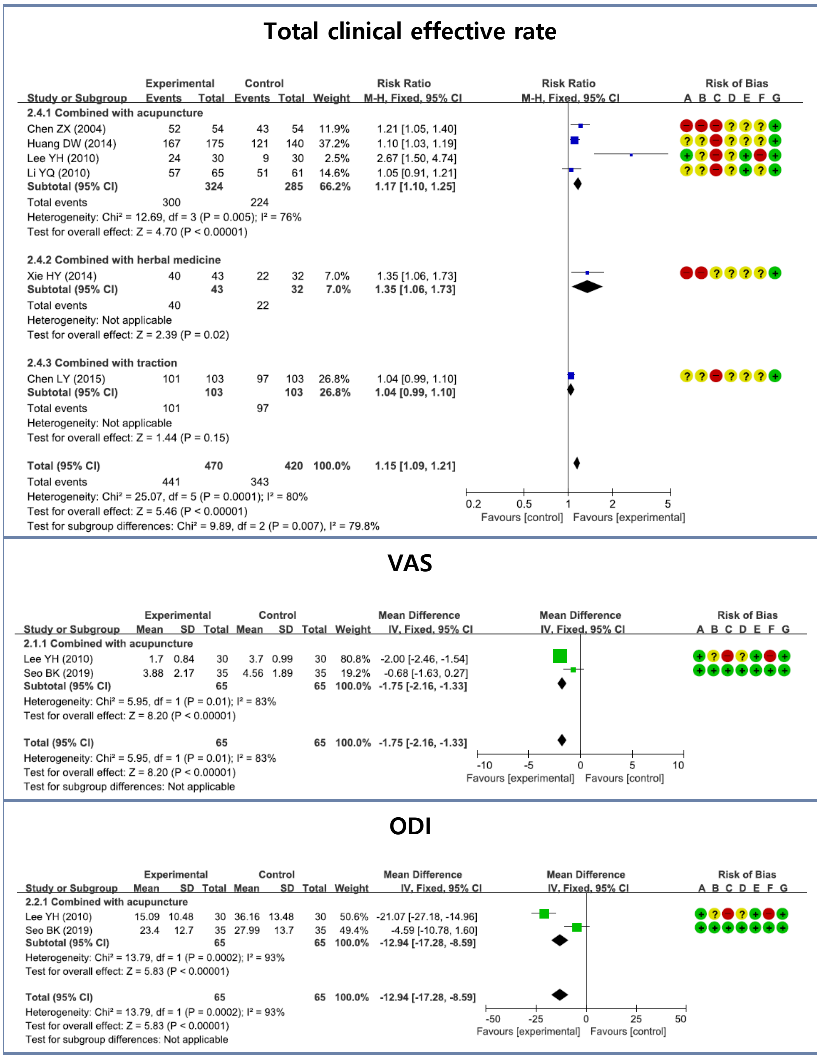

JOA

Study or Subgroup	Experimental Mean	Experimental SD	Experimental Total	Control Mean	Control SD	Control Total	Weight	Mean Difference IV, Fixed, 95% CI
**2.3.1 Combined with acupuncture**								
Lee YH (2010)	25.67	3.29	30	23.3	3	30	100.0%	2.37 [0.78, 3.96]
**Subtotal (95% CI)**			**30**			**30**	**100.0%**	**2.37 [0.78, 3.96]**
Heterogeneity: Not applicable								
Test for overall effect: Z = 2.92 (P = 0.004)								
**Total (95% CI)**			**30**			**30**	**100.0%**	**2.37 [0.78, 3.96]**
Heterogeneity: Not applicable								
Test for overall effect: Z = 2.92 (P = 0.004)								
Test for subgroup differences: Not applicable								

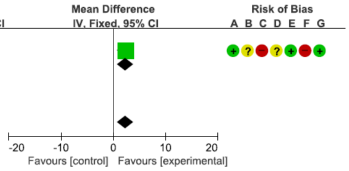

Cupping + usual conventional therapy vs. usual conventional therapy

Total clinical effective rate

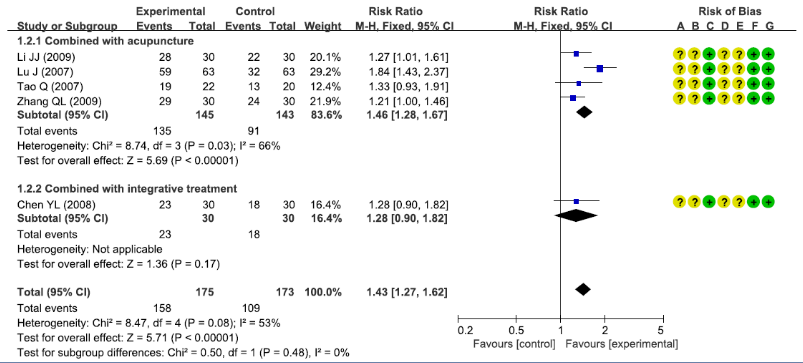

VAS

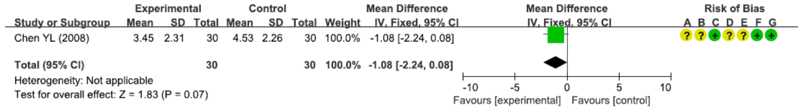

Supplement: Supplementary file 1 [file healthcare-10-00246-s001.zip › Supplementary S4(forest flot).pdf]
